# The impact of the COVID-19 pandemic on cardiovascular disease prevention and corresponding geographical inequalities in England: interrupted time series analysis

**DOI:** 10.1186/s12889-023-17282-3

**Published:** 2023-12-07

**Authors:** Alejandra Castanon, Katja Grasic, Simon Chen, Florence Ma, Godspower Oboli, Benjamin D. Bray, Andrew Hughes, Martin White, Shahed Ahmad, Jonathan Pearson-Stuttard

**Affiliations:** 1 Health Analytics, Lane Clark & Peacock LLP, 95 Wigmore Street, London, W1U 1DQ UK; 2 Office for Health Improvement and Disparities (OHID), Department of Health and Social Care, 39 Victoria Street, London, SW1H 0EU UK; 3 NHS England, 133-155 Waterloo Rd, London, SE1 8UG UK

**Keywords:** COVID-19 impact, Cardiovascular disease, Health inequalities, Hypertension, Atrial fibrillation

## Abstract

**Background:**

There has been disruption to the detection and management of those with hypertension and atrial fibrillation (AF) during the COVID-19 pandemic. This is likely to vary geographically and could have implications for future mortality and morbidity. We aimed to estimate the change in diagnosed prevalence, treatment and prescription indicators for AF and hypertension and assess corresponding geographical inequalities.

**Methods:**

Using the Quality and Outcomes Framework (2016/17 to 2021/22) and the English Prescribing Datasets (2018 to 2022), we described age standardised prevalence, treatment and prescription item rates for hypertension and AF by geography and over time. Using an interrupted time-series (ITS) analysis, we estimated the impact of the pandemic (from April 2020) on missed diagnoses and on the percentage change in medicines prescribed for these conditions. Finally, we described changes in treatment indicators against Public Health England 2029 cardiovascular risk targets.

**Results:**

We observed 143,822 fewer (-143,822, 95%CI:-226,144, -61,500, *p* = 0.001) diagnoses of hypertension, 60,330 fewer (-60,330, 95%CI: -83,216, -37,444, *p* = 0.001) diagnoses of AF and 1.79% fewer (-1.79%, 95%CI: -2.37%, -1.22%), *p* < 0.0001) prescriptions for these conditions over the COVID-19 impact period. There was substantial variation across geography in England in terms of the indirect impact of the COVID-19 pandemic on the diagnosis, prescription, and treatment rates of hypertension and AF. 20% of Sub Integrated Care Boards account for approximately 62% of all missed diagnoses of hypertension. The percentage of individuals who had their hypertension controlled fell from 75.8% in 2019/20 to 64.1% in 2021/22 and the percentage of individuals with AF who were risk assessed fell from 97.2% to 90.7%.

**Conclusions:**

Hypertension and AF detection and management were disrupted during the COVID-19 pandemic. The disruption varied considerably across diseases and geography. This highlights the utility of administrative and geographically granular datasets to inform targeted efforts to mitigate the indirect impacts of the pandemic through applied secondary prevention measures.

**Supplementary Information:**

The online version contains supplementary material available at 10.1186/s12889-023-17282-3.

## Introduction

The COVID-19 pandemic has indirectly impacted the burden of noncommunicable diseases in the population by impacting diagnosis and management. While cardiovascular disease (CVD) excess mortality has been persistently high during 2022 [1], it is likely that nonfatal outcomes have also been impacted. Indeed, a recent publication found that almost 500,000 individuals missed out on antihypertensive medications in England, Wales and Scotland between March 2020 and July 2021 [2].

Hypertension and atrial fibrillation (AF) are major drivers of premature death and morbidity in the UK and worldwide. Elevated blood pressure (BP) significantly increases the risk of heart, brain, kidney, and other diseases [3]. AF is the most common cardiac arrhythmia [4]. Individuals with AF are 5 times more likely to suffer a stroke and for that stroke to be severe [5]. Both conditions however can be effectively managed to reduce this risk.

Treatment with anticoagulants substantially reduces the risk of stroke. Patients with pre-existing AF should have their risk of stroke per year estimated using the CHA2DS2-VASc score. A CHA2DS2-VASc score of 0 is considered "low-risk" and anticoagulation is not recommended. Oral anticoagulation is recommended in patients with AF and an elevated CHA2DS2-VASc score of 2 or more [6]. Similarly treatment with antihypertensives reduces the risk of heart attack and stroke among other health problems [3].

Public Health England’s (PHE) 10-year CVD ambition in 2019 was to increase the percentage of individuals with hypertension diagnosed from 57 to 80% and the percentage of patients with hypertension whose BP is controlled from 56 to 80% by 2029 [7]. The respective targets for AF include increasing the number of people with AF detected from 79 to 85% and the percentage of those known to be at high risk for stroke to be adequately anticoagulated from 84 to 90%. By March 2020 although targets for hypertension had not been achieved those for AF had been exceeded.

Disruption in the detection and management of those with hypertension and AF, and how this varies geographically is likely to have implications for future mortality and morbidity. These data could help target prevention resources more effectively. We therefore aimed to estimate the change in prevalence, treatment and prescription indicators for AF and hypertension during the COVID-19 pandemic impact period and assess corresponding geographical inequalities.

## Methods

### Setting and data sources

We combined several publicly available data sources (Quality and Outcomes Framework (QOF), English Prescribing Dataset (EPD) and Office for National Statistics (ONS) deprivation data) to estimate diagnosis, prescription and treatment rates at varying geographical levels across England.

Hypertension and AF prevalence and corresponding treatment achievement rates were identified via QOF data, covering the period from April 2015 to March 2022 [8]. QOF is a voluntary annual reward and incentive programme for all general practitioner (GP) practices in England whose aim is appropriate resourcing and rewarding good practices.

Prescription data of medications prescribed and dispensed in the community were obtained from the EPD [9] sourced from the National Health Service Business Services Authority (NHS BSA) Open Data Portal for prescriptions issued in England and dispensed in Great Britain, Guernsey, Alderney, Jersey, and the Isle of Man from April 2018 to April 2022. Data prior to 2018 were not used because sensitivity analysis showed erratic prescription rates in 2016 and 2017 likely related to CCG boundary changes. Number of monthly prescription items was aggregated at Clinical Commissioning Group (CCG) [10] level as presented in EPD.

The ONS index of multiple deprivation (IMD) dataset [11] for small geographical areas was used to map IMD at lower layer super output area (LSOA) level.

Data were extracted for the following QOF indicators for each calendar year at the GP practice level: hypertension prevalence (QOF indicator HYP001), AF prevalence (QOF indicator AF001), BP control in those under age 80 defined as ≤ 140/90 (QOF indicator HYP003), BP control in those aged 80 and older defined as ≤ 150/90 (QOF indicator HYP007), the percentage of AF patients with a CHA2DS2-VASc score of 2 or more who are currently treated with anti-coagulant drug therapy (AF007) and the percentage of patients with AF in whom stroke risk has been assessed using the CHA2DS2-VASc score risk stratification scoring system in the preceding 12 months (excluding those patients with a previous CHADS2 or CHA2DS2-VASc score of 2 or more) (AF006). Note that neither AF indicator was reported in 2020/21.

We examined prescription rates of medications used to treat hypertension and heart failure as a proxy for CVD management. Cardiovascular medications were identified according to the legacy British national formulary (BNF) hierarchy [12]. We identified relevant paragraphs in chapter 2 of the BNF to identify medications indicated for hypertension only and heart failure only, and indicated for both conditions (Table S1 in Supplement). Data on oral-anticoagulants were extracted from subparagraph 2.8.2.

### Geographical mapping and population rates

Hypertension and AF prevalence and treatment indicators were aggregated from the GP practice level to the Sub Integrated Care Board (sub-ICB), regional and national levels using QOF data from April 2019 to March 2020 (2019/20) and separately using QOF data from April 2021 to March 2022 (2021/20). The QOF data were further used to map GP practice level data to the LSOAs. To determine the population registered in each GP practice, patient registration data from the Primary Care Registration database within the National Health Application and Infrastructure Services (NHAIS) were used [13].

GP practice registration data was mapped to LSOA level using two assumptions: i) the entire population within an LSOA is registered with a GP practice and ii) prevalence estimates applied to the whole registered population of a GP practice, regardless of which LSOA they live in. These assumptions lead to over- and under-estimations of prevalence particularly at small geographical level (Supplementary Methods 1.1) which are reduced at larger (e.g., sub-ICB) geography.

Geographical mapping was performed using shapefiles provided by the ONS. Over the duration of the analysis, NHS England CCGs were restructured as sub-ICBs which along with changes to the administrative naming of these boundaries slightly modified the geographical boundaries (Supplementary Methods 1.2).

To explore progress against PHE CVD targets the differences between observed treatment achievement rates in 2021/22 and the target rates for AF and hypertension (80% patients with hypertension to have their BP controlled and 90% of individuals with AF known to be at high risk for stroke to be adequately anticoagulated) were estimated at GP practice level and mapped to other geographies as described above.

Age specific hypertension QOF treatment indicators were pooled by estimating at GP practice level the ratio of the number of patients with hypertension whose BP was controlled (i.e. HYP003, n1 and HYP007, n2) to the total number of patients in the hypertension register (N) using the formula: ∑ [(n1i + n2i)/N], where i represents each individual GP practice.

To enable meaningful comparisons between geographies, we age and sex adjusted prevalence estimates using an indirect standardisation approach (Supplementary Methods 1.3).

Prescription data were aggregated and mapped to 2021 CCGs, considering mergers over time using CCG codes publicly available from NHS and ONS. CCG population was based on 2020 mid-year estimates from ONS [14].

A summary of the data sources, calendar years available and underlying population estimates can be found in Table S2.

### Statistical analysis

Main outcomes were 1) age standardised prevalence, treatment indicators and prescription item rates for hypertension and AF by geography; 2) how they change over time and a comparison to what we would expect in the absence of the pandemic and 3) on progress towards 10-year CVD ambitions. As a secondary outcome we investigated the relationship between hypertension and AF prevalence and corresponding treatment indicators with index of multiple deprivation of local area.

To estimate the indirect impact of the COVID-19 pandemic on hypertension and AF prevalence and on the rate of medicine dispensations, we performed an interrupted time series analysis (ITS). Due to the lack of documentation on GP practice closures and merges over time, along with difficulties obtaining precise modelling estimates at the small geography level, the analysis was performed at sub-ICB level.

In the first step, we conducted an interrupted time series analysis on the pre- and post-COVID data, which captures both the shift in levels and the shift in the trend of the outcome. Since each sub-ICB follows a different trend and starts at a different slope, we employed a random effects (mixed-effects) model, allowing for distinct trends. By allowing for individual trajectories, for each sub-ICB, we account for the fact that those with extreme initial measurements might have unique trajectories. The Model was:

$${Y}_{it} = {\beta }_{0} + bet{a}_{1} {T}_{it} + bet{a}_{2} COVI{D}_{it} + bet{a}_{3}COVI{D}_{it}{T}_{it}+{b}_{0i} + {b}_{1i} {T}_{it}+ {\epsilon }_{it}$$

Where ${T}_{it}$ is the outcome for sub-ICB I at time t. T is a variable representing time, which is measured either in months or years, depending on the outcome in question. COVID is a dummy variable, taking a value of 0 in the pre-COVID period and 1 in the post-COVID period. B_0i_ is the random intercept for sub-ICB_i_, while ${b}_{1i} {T}_{it}$ is the random slope effect for sub-ICB_i_ which captures the deviation of the trend for sub-ICB_i_ from the average trend. ${\epsilon }_{i}t$ is the error term. In this model the key coefficients measuring the impact of COVID are beta_2_ and beta_3_: beta_2_ represents the average level effect of COVID across all sub-ICB units, while beta_3_ captures the changes in trend in the post-covid period. To account for seasonality, we further include in the prescription analysis only, a vector of dummy variables indicating calendar months.

We utilise a distinct estimation model to forecast the cumulative impact of the pandemic on outcomes at the sub-ICB level. For this approach, we conduct the regression solely on the pre-COVID data. This aids in predicting the anticipated value of specific outcomes, assuming the trend had remained constant. In this case, we use the following regression model with the description of the coefficients identical to those used in the ITS described above:

$${Y}_{it} = {\beta }_{0} + bet{a}_{1} {T}_{it} +{b}_{0i} + {b}_{1i} {T}_{it}+ {\epsilon }_{it}$$

Using the coefficients from this model, we predict that the estimated outcomes had the same trend.

Estimated missed diagnoses were computed as the difference between the estimated counterfactual prevalence and the observed prevalence, and presented as absolute numbers and as rates per 100,000 population over the first year of the pandemic. Prescription data are presented as monthly prescription item rates per 1,000 person days. An average monthly prescription rate was calculated over the whole pandemic period. Percentage difference from the expected rate/prevalence was calculated using the formula: (expected– observed) / expected. To calculate the confidence interval (CI) of the difference between the observed and expected values, we assumed that the values were independent. CI were derived from the standard error (SE) of the difference (calculated as the square root of the sum of the squares of SE for the individual components). Using the same ratio as that published by Dale, et al. [2] (based on the National Institute of Health and Care Excellence (NICE) hypertension treatment model) we estimated additional CVD events due to untreated hypertension (Supplementary Methods 1.4).

Nationally a total of 25 GP practices (0.4% of 6,720) did not submit hypertension treatment data to QOF in 2019/20 and 25 (0.4% of 6468) in 2021/22 and were excluded from the analyses. A further 48 GP practices were removed in 2021/22 as they contained no age distribution data, and thus age standardisation could not be performed.

We developed an online visualisation platform to enable a more comprehensive exploration of the results which is hosted at (https://cvdrapidanalysis.lcp.com/). Data management and analyses were conducted in R version 4.2.2.

## Results

### Prevalence of hypertension and atrial fibrillation

**Fig. 1:**
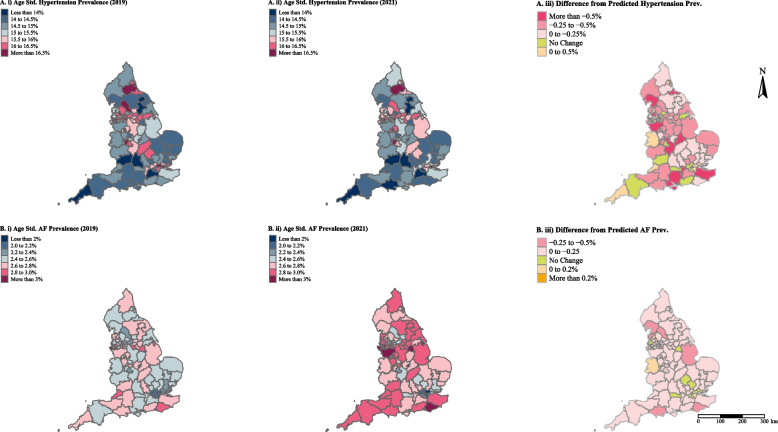
Sub-ICB age standardised prevalence of **A**. hypertension and **B**. atrial fibrillation for i). 2019/20, ii). 2021/22 and ii). difference between observed and expected prevalence for 2021/22

The age standardised prevalence rate of diagnosed hypertension in England was 15.55% (14.10% crude) between 2019/20, decreasing to 15.11% (13.97% crude) in 2021/22. This varied across regions (Fig. 1Aii) with the highest prevalence for 2021/22 in the North East and Yorkshire (15.64%), followed by North West (15.60%), Midlands (15.54%), London (15.19%), East of England (14.77%), South East (14.44%). The lowest prevalence was observed in the South West (14.23%). At sub-ICB level age standardised prevalence rates of diagnosed hypertension ranged from 17.71% in Staffordshire and Stroke-on-Trent sub-ICB to 12.17% in NHS Sussex sub-ICB (Table S3).

Age standardised prevalence rate of diagnosed AF in England was 2.55% (2.05% crude) in 2019/20, increasing to 2.66% (2.09% crude) in 2021/22. London had the lowest prevalence (2.08%) of AF in 2021/22 and the South West (2.90%) had the highest (Fig. 1Bii). At sub-ICB level age standardised prevalence ranged from 3.32% in Cheshire and Merseyside sub-ICB to 1.91% in North East London sub-ICB (Table S4).

At LSOA level age standardised prevalence ranged from 6.94% to 22.13% for hypertension and from 1.10 to 4.15% for AF (Tables S5 and S6).

### Estimated missed hypertension and AF diagnoses during the COVID-19 pandemic

The overall effect of COVID-19 on hypertension prevalence was a decrease of 0.54 percentage points (*p* = 0.127). In addition, we observed an increase in the prevalence rate in the post-COVID period, with annual prevalence rates increasing by 0.047 percentage points per year (*p* = 0.07) (Table S7 and Figure S1). Assuming that the pre-pandemic trends would continue during the pandemic period, the estimated crude national prevalence of hypertension in March 2021 would have been 14.43% (95%CI:14.10, 14.76, *p* = 0.001). The difference between the observed and the modelled crude prevalence rates indicates that an estimated 143,822 fewer (-143,822, 95%CI:-226,144, -61,500, *p* = 0.001) individuals in England (-233.00, 95%CI:-366.36, -99.63 per 100,000 population) were diagnosed with hypertension over the COVID-19 impact period (April 2020 to March 2021). This represents 3.19% of prevalent cases. The Midlands had the greatest number of missed diagnoses (38,299 fewer, *p* = 0.246) however the North West region had the highest estimated number of missed diagnosis when adjusted for population size (-359.3, 95%CI:-972, 253 per 100,000 population, *p* = 253). London was the only region to report more diagnoses than expected (2,669, *p* > 0.5), (Table S8). The 20% of sub-ICBs with the greatest estimated number of missed hypertension diagnosed accounted for approximately 62% of all missed diagnoses (Figs. 1Aiii and 2).

**Fig. 2:**
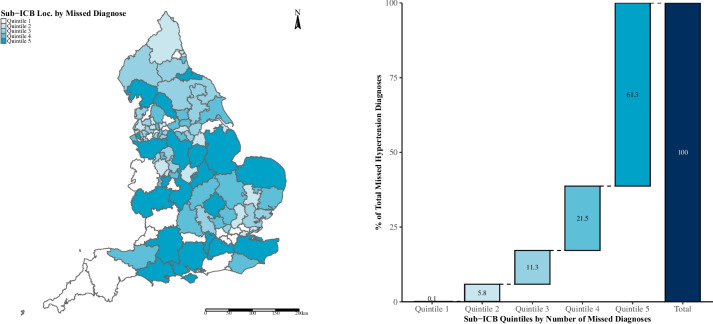
Missed diagnoses of hypertension (at Sub-ICB level) between April 2020 and March 2021, stratified by quintile

The overall effect of COVID on AF prevalence was an increase of 0.073 percentage points (*p* = 0.056) due to the onset of COVID, followed by a decrease in the annual prevalence rate, decreasing by 0.036 percentage points (*p* = 0.001)per year, in the post-COVID period (Table S7 and Figure S2). The predicted national prevalence in March 2021 was 2.15% (95%CI: 2.05%, 2.27%, *p* = 0.001). An estimated 60,330 fewer (-60,330, 95%CI: -83,216, -37,444, *p* = 0.001) individuals in England (-97.78, 95%CI: -134.87, -60.69 per 100,000 population, *p* < 0.0001) were diagnosed with AF over the COVID-19 impact period representing 2.79% of prevalent cases. The 20% of sub-ICBs with the greatest number of missed AF diagnoses accounted for approximately 54% of all missed diagnoses during this period (Figure S3, Fig. 1Biii).

### Treatment indicators

**Fig. 3:**
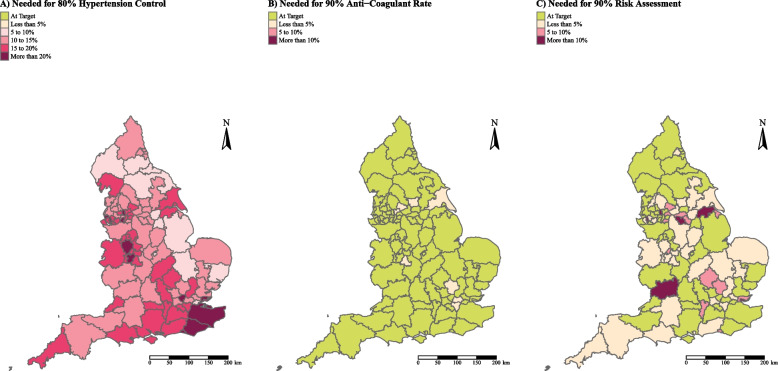
Sub-ICB treatment achievement rates in 2021/22 benchmarked against 2029 cardiovascular risk targets for **A** Hypertension, **B** individuals with a CHA2DS2-VASc score of 2 or more treated with anti-coagulation therapy and **C** percent of patients with AF who are risk assessed

We found a stark decrease in the percentage of patients with diagnosed hypertension for whom the condition was adequately controlled (BP ≤ 140/90 if aged < 80y or BP ≤ 150/90 if ≥ 80y). In England 75.77% of individuals with hypertension had their condition controlled in 2019/20, decreasing by 11.06 percentage points to 64.71% in 2021/22. By 2021/22, no sub-ICB (out of 106 sub-ICBs) had achieved the 80% treatment target set for hypertension treatment indicators by 2022 (Fig. 3A) and 50 sub-ICBs were achieving 65% or less (Table S9). At a regional level the South East had the lowest treatment achievement rate (61.08%) followed by London (63.09%). The North East and Yorkshire had the highest treatment achievement rate (68.82%), (Table S10).

As of 2021/22, all but 8 sub-ICB in England were treating at least 90% of individuals with a CHA2DS2-VASC score of 2 or more with oral anticoagulants (Fig. 3B). The sub-ICBs that did not achieve the 90% treatment target all achieved at least 85%. We did not find evidence of a difference in achievement rates between 2019/20 and 2021/22: 91.71% of individuals with AF and a CHA2DS2-VASc score of 2 or more were on anticoagulants in 2019/20 and 91.85% in 2021/22, (Figure S4). However, the number of sub-ICBs meeting the risk assessment targets (i.e. > 90%) in 2021/22 was much lower (51% meeting target), with 6 sub-ICBs risk assessing less than 80% of individuals with AF (Fig. 3C). Furthermore, there was a decrease in the percent of individuals risk assessed from 97.18% in 2019/20 to 89.04% in 2021/22 (Figure S2). At a regional level, the East of England had the lowest percent of individuals risk assessed (86.84%), followed by, South West (87.75%), North East and Yorkshire (87.91%), Midlands (89.45%), London (89.46%), North West (89.96%), with the South East having the highest percent risk assessed (90.82%).

The variation in the treatment achievement rates by LSOAs was substantial, ranging from 27.32% to 93.39% for hypertension and from 8.75% to 100% for percent of individuals with AF who were risk assessed (Table S11 and Table S12).

### Prescription data

**Fig. 4:**
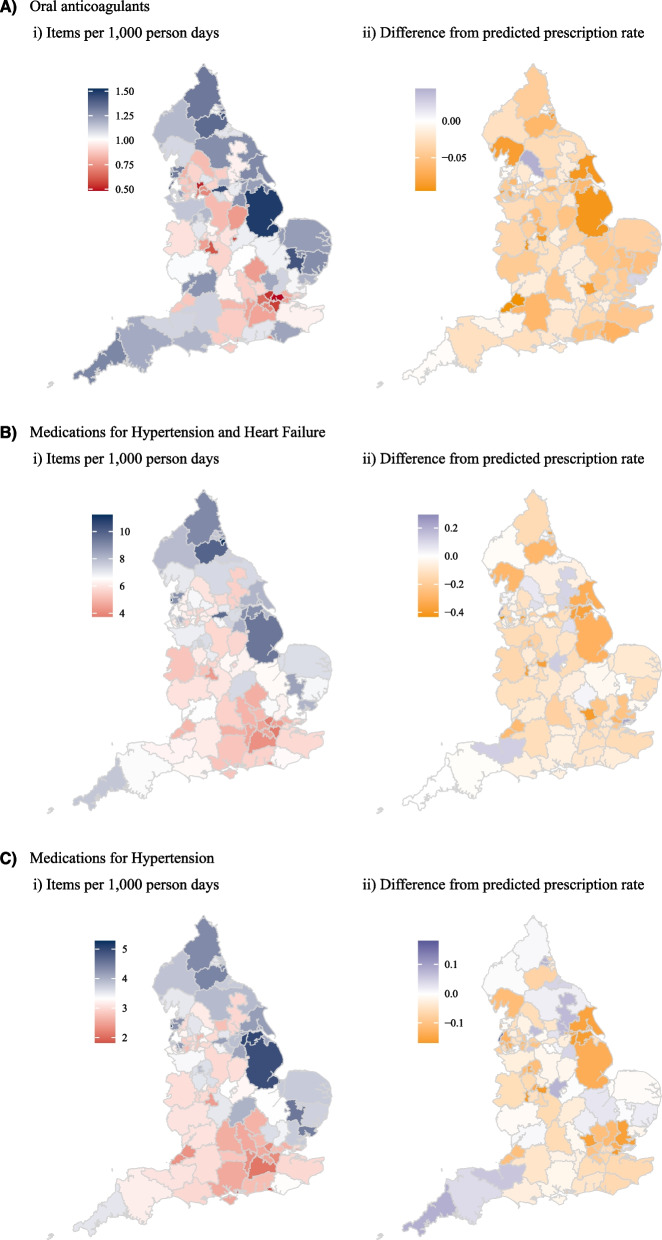
Sub-ICB level average monthly prescription items per 1,000 person days (i) and difference between observed and expected prescription rate (ii) between March 2020 and April 2022 for **A** Oral anticoagulants, **B** Medications for hypertension and heart failure and **C** Medications for hypertension only

We found an initial increase in prescription rates across all antihypertensive, heart failure and oral anticoagulant medications in March 2020, followed by a drop in the prescription rate (Table S7 and Figure S5). Whereas most of the sub-ICBs prescribed fewer oral anticoagulants after the start of the COVID-19 pandemic, the results were variable for hypertension and heart failure medications (Fig. 4Bii and Cii). Between March 2020 and April 2022 the percentage difference in prescription rates for all included medications was 1.79% lower than expected (-1.79%, 95%CI: -2.37%, -1.22%, *p* < 0.0001), for oral anticoagulants, it was on average 3.51% lower than expected based on pre-pandemic trends (-3.51%, 95%CI: -4.21%, -2.82%, *p* < 0.0001). Difference in rates was -1.76% (95%CI: -2.33%, -1.19%, *p* < 0.0001) for hypertension and heart failure drugs, -1.34% (95%CI: -1.91%, -0.77%, *p* < 0.0001) for hypertension only drugs and -2.81% (95%CI: -3.57%, -2.05%, *p* < 0.0001) for heart failure only drugs, Table 1.

**Table 1:** Average monthly prescription rate (items per 1,000 person days) and percentage difference in prescription rates from March 2020 – April 2022

Medication	Heart Failure		Hypertension and Heart Failure		Hypertension		Oral anticoagulants		Total	
Region	Prescription rate	Percent difference (95%CI; p-value)^a^	Prescription rate	Percent difference (95%CI; p-value)^a^	Prescription rate	Percent difference (95%CI; p-value)^a^	Prescription rate	Percent difference (95%CI; p-value)^a^	Prescription rate	Percent difference (95%CI; p-value)^a^
East of England	0.28	-4.05% (-6.69%, -1.41%; 0.003)	6.27	-1.77% (-3.06%, -0.49%; 0.007)	3.37	-1.32% (-2.49%, -0.15%; 0.027)	1.05	-3.75% (-4.95%, -2.55%; < 0.0001)	10.97	-1.9% (-3.06%, -0.74%; 0.001)
London	0.21	-2.3% (-4.08%, -0.52%; 0.011)	4.28	-3.02% (-3.95%, -2.1%; < 0.0001)	2.51	-3.05% (-3.71%, -2.39%; < 0.0001)	0.59	-3.9% (-5.26%, -2.54%; < 0.0001)	7.58	-3.07% (-3.91%, -2.23%; < 0.0001)
Midlands	0.32	-3.16% (-4.48%, -1.85%; < 0.0001)	6.07	-1.92% (-2.72%, -1.13%; < 0.0001)	3.30	-1.36% (-2.21%, -0.52%; 0.002)	0.90	-3.95% (-5.16%, -2.74%; < 0.0001)	10.59	-1.97% (-2.76%, -1.18%; < 0.0001)
North East and Yorkshire	0.46	-1.99% (-3%, -0.98%; 0.0001)	7.53	-1.02% (-1.77%, -0.28%; 0.007)	3.68	-0.36% (-1.05%, 0.33%; 0.303)	1.11	-2.56% (-3.54%, -1.58%; < 0.0001)	12.79	-1.03% (-1.72%, -0.34%; 0.004)
North West	0.35	-2.05% (-3.36%, -0.73%; 0.002)	6.65	-1.68% (-2.42%, -0.93%; < 0.0001)	3.32	-1.58% (-2.32%, -0.84%; < 0.0001)	0.96	-3.39% (-4.28%, -2.5%; < 0.0001)	11.28	-1.82% (-2.51%, -1.14%; < 0.0001)
South East	0.18	-0.97% (-2.72%, 0.78%; 0.28)	5.23	-1.86% (-2.31%, -1.4%; < 0.0001)	2.63	-1.63% (-2.25%, -1.02%; < 0.0001)	0.92	-3.54% (-4.77%, -2.31%; < 0.0001)	8.97	-1.95% (-2.42%, -1.49%; < 0.0001)
South West	0.28	-2.19% (-5.16%, 0.77%; 0.15)	6.33	-1.3% (-2.67%, 0.07%; 0.062)	3.01	-0.37% (-1.94%, 1.21%; 0.649)	1.13	-3.52% (-5.61%, -1.42%; 0.001)	10.75	-1.31% (-2.76%, 0.14%; 0.078)
England	0.30	-2.81% (-3.57%, -2.05%; < 0.0001)	6.00	-1.76% (-2.33%, -1.19%; < 0.0001)	3.11	-1.34% (-1.91%, -0.77%; < 0.0001)	0.94	-3.51% (-4.21%, -2.82%; < 0.0001)	10.34	-1.79% (-2.37%, -1.22%; < 0.0001)

^a^Monthly percentage difference of prescription rate was calculated using the formula: (observed rate – expected rate) / expected rate × 100%, which was then averaged over March 2020 – April 2022

The Midlands had the greatest percentage difference in prescription rate for oral anticoagulants (-3.95%, 95%CI: -5.16%, -2.74%, *p* < 0.0001), while the North East and Yorkshire was least impacted (-2.56%, 95%CI: -3.54%, -1.58%, *p* < 0.0001). At CCG level the percentage difference in prescriptions ranged from -9.75% to 5.46%, (Fig. 4Aii and Table S13).

For hypertension and heart failure medication (the largest group of antihypertensive medications), London had the greatest percentage difference in prescription rate (-3.02%, 95%CI: -3.95%, -2.1%, *p* < 0.0001) and North East and Yorkshire the least (-1.02%, 95%CI: -1.77%, -0.28%, *p* = 0.007). At a sub-ICB level the percentage difference in prescriptions ranged from -7.11% to 4.05%, (Table S14).

### Association with deprivation of local area

Age standardised prevalence of hypertension in LSOAs in the most deprived decile was 13.55% compared to 14.16% in the least deprived decile (*p* < 0.0001). Similar figures for AF were 2.65% and 2.78% respectively (*p* < 0.0001), (Figure S6). No other significant associations were found (Figures S6-S7).

### Impact on cardiovascular events

We estimated that missed diagnoses of hypertension over the first year of the pandemic could lead to approximately 4,073 CVD events including 1,036 strokes and 680 MIs over a lifetime. However, if these individuals are diagnosed and treated over the next 5 years 3,263 CVD events could be averted, including 898 strokes and 463 MIs. There were large regional variations in this with no additional events reported in London while 27% of additional CVD events were in the Midlands.

## Discussion

Using data on prevalence, treatment indicators, and prescriptions as a proxy for management of hypertension and atrial fibrillation from publicly available datasets we investigated the impact of the COVID-19 pandemic on diagnosis and management of these chronic conditions alongside corresponding geographical inequalities in England. There are several key findings from our analysis; substantially fewer new diagnoses than would have been expected, particularly in hypertension; substantial disruption to management of the prevalent population and disruption to the community based prescription of CVD preventative medications. This analysis delivers useful and actionable information from publicly available data avoiding the need for patient-level repositories.

First, 143,822 fewer people were diagnosed with hypertension during the first year of the pandemic than might have been expected in absence of the pandemic. This equated to 3.19% of prevalent cases. The variation in missed diagnoses at the local level is demonstrated by 20% of sub-ICBs accounting for 62% of all missed hypertension diagnoses during this period. There were far fewer missed AF diagnoses during this time (*n* = 60,330 or 2.79% of prevalent cases).

Second, treatment indicators in those with established conditions, representing effective management, were significantly disrupted. Those with diagnosed hypertension who were well controlled dropped from 75.77% to 64.71% across England, with the South East region having the lowest attainment. In AF, while the percent of individuals with raised CHA2DS2-VASc scores who were on anticoagulants remained high, the percentage of individuals with AF who underwent risk assessments decreased. This fell from 97.18% to 89.04% nationally, with even lower rates observed in several sub-ICBs.

Third, we found disruption in the prescription of CVD preventative medications. The prescription of oral anticoagulants was particularly impacted with 3.51% less prescriptions than expected in the first two years of the pandemic. As with other outcomes, rates varied significantly by geography.

These disruptions are likely to impact clinical outcomes (fatal and non-fatal) but the time periods for doing so will vary and are difficult to determine. Our findings can be partially explained by the reduction in the number of GP consultations and in A&E visits following the start of the pandemic [15, 16]. Changes in prevalence cannot solely be attributed to reduced diagnoses, since the period under study was one with higher than usual mortality [17]. Although population denominators should adjust with time those used in this analysis may not have fully done so. Missed diagnoses of AF should be interpreted with caution since age adjusted prevalence increased (2.55% vs 2.66%) and once the 85% target was achieved, a decrease in the detection rate was expected.

The Midlands is identified as a particularly badly affected region across most indicators, while North East and Yorkshire had consistently relatively good performance. Although we estimate no missed diagnosis of hypertension in London, prescription data suggest significant impact on hypertension control, backed by the low treatment rates in the city. This could be driven by people leaving the city during lockdown and having their medication prescribed elsewhere. Prescription data may also be impacted by increases in hospitalised patients shifting prescribing from the community to the hospital setting.

Other publications have explored the impact of the pandemic on the prescription of CVD preventative medication in the United Kingdom [2, 18, 19]. Studies are consistent with our results by finding a decrease in diagnoses and prescribing in the post-pandemic period but are limited in that they do not consider data post July 2021 and none present small area statistics. Our study extends results by Dale et al., [2] which suggest that there was a substantial drop (approximately 500,000) in the number of individuals initiating management of different cardiovascular conditions in the UK, including type 2 diabetes mellitus, hypertension, hypercholesterolemia and atrial fibrillation. This could potentially result in over 13,000 additional CVD events [20]. This study’s lower figure of just under 210,000 is limited to individuals missing out on antihypertensive and oral-anticoagulant medication. Results between studies are not directly comparable since we based our estimates on prevalence rather than new prescription items and limited our analysis to hypertension and AF diagnosed in England. We did not use prescriptions to estimate the number of individuals who missed out on antihypertensive medication because the English prescribing dataset does not distinguish between incident, prevalent and multiple prescriptions.

The ability of the ITS design to support causal inferences is strengthened when the intervention (in our study, the impact of COVID-19 related lockdowns) is abrupt and when changes in the population or other external factors that could be responsible for changes in trends can be excluded [21]. To mitigate bias from extrapolations of prevalence rates to small geographies, ITS analysis was carried out at sub-ICB level. We note that at lower geographical level results are non-statistically significant, however for public health interventions lack of statistical significance does not preclude the need for population level interventions. Furthermore we did not disaggregate oral anticoagulants in our analysis because pre-COVID, patients had been transitioning from warfarin to direct oral anticoagulants (DOACs) and during the pandemic national clinical guidance [22] recommended switching patients to DOACs to minimise monitoring and reduce the requirement for physical attendance which will influence trends in prescribing over the study period [18]. 

The evaluation of treatment indicators at small geography enables the identification of areas of most unmet need allowing timely targeted intervention, which prevalence and prescription rates are also less amenable. However, changes in treatment achievement rates may be due to the disruption to recording of blood pressure readings by GP practices rather than poor management per se given CVDPREVENT results suggest little change in prescribed antihypertensive medication between 2019/20 and 202/21 [23]. While ensuring that those with raised CHA2DS2-VASc scores remained on anticoagulants was a protected indicator in QOF throughout the pandemic (i.e. payments were made to practices irrespective of activity recorded), risk assessment was not.

Our analysis is limited to routinely published aggregated data. Submitting data to QOF is voluntary and therefore is not comprehensive (although data are collected from 97.5% of all GPs in England) [24] and it does not include breakdown by sex or age. We were able to age and sex adjust at GP practice level using other published data but not for other factors (i.e. socioeconomic deprivation).

Outliers at LSOA level should be interpreted with caution given the potential for over/underestimation of prevalence in small area estimates. However, it is these statistics that are most helpful for policymakers to implement targeted action and on balance are of public health relevance.

Geographical health disparities are a top agenda priority for policy makers and small area statistic are vital tools to enable targeted action and resources [25, 26]. This study found that the management of CVD was substantially impacted during the pandemic. There were material geographical inequalities that are likely to be reflected in worsening cardiovascular outcomes in years to come if action is not taken. By providing small-area estimations of the pandemic's indirect impact on CVD this study can support policymakers designing targeted approaches to addressing unmet need providing best value for money.

### Supplementary Information

**Additional file 1:** Supplemental methods. **Table S1.** List of paragraphs from BNF chapter 2 according to clinical indication. **Table S2.** Summary of data sources, calendar years and population used by analysis and disease. **Table S3.** Sub-ICB age standardised hypertension prevalence. Top 10 Sub-ICBs with the highest and lowest prevalence. **Table S4.** Sub-ICB age standardised atrial fibrillation prevalence. Top 10 Sub-ICBs with the highest and lowest prevalence. **Table S5.** LSOAs age standardised hypertension prevalence. Top 10 LSOAs with the highest and lowest prevalence. **Table S6.** LSOAs age standardised atrial fibrillation prevalence. Top 10 LSOAs with the highest and lowest prevalence. **Table S7.** Results of the overall ITS analysis on the Sub-IBC level. **Table S8.** Missed diagnoses of hypertension and impact on cardiovascular disease by Region. **Table S9.** Sub-ICBs hypertension treatment achievement rates. Top 10 sub-ICBs with the highest and lowest achievement rates. **Table S10.**  Hypertension treatment achievement rate (2021-22) by Region. **Table S11.** LSOAs hypertension treatment achievement rates. Top 10 LSOAs with the highest and lowest achievement rate. **Table S12.** LSOAs Atrial Fibrillation risk assessment treatment achievement rate. Top 10 LSOAs with the highest and lowest achievement rates. **Table S13.** CCGs percent difference in monthly prescription rates of oral anticoagulants medication between March 2020 – April 2022. Top 10 CCGs with the highest and lowest percentage difference. **Table S14.** CCGs percent difference in monthly prescription rates of hypertension and heart failure medication between March 2020 – April 2022. Top 10 CCGs with the highest and lowest percentage difference. **Figure S1.** Observed and predicted prevalence of hypertension pre and post COVID-19 in England. **Figure S2.** Observed and predicted prevalence of atrial fibrillation pre and post COVID-19 in England. **Figure S3.** Age standardised prevalence rates of A) Hypertension Figure S3. Missed diagnoses of atrial fibrillation between April 2020 and March 2021. **Figure S4.** Treatment achievement rates in 2019/20 and 2021/22 at sub-ICB level in England for A) Hypertension, B) individuals with a CHA2DS2-VASc score of 2 or more treated with anti-coagulation therapy and C) AF risk assessment. **Figure S5.** Prescription rate (items per 1,000 person days) for medications for A) Heart failure, B) Hypertension, C) both Hypertension and Heart Failure and D) Oral anticoagulants pre and post COVID-19. **Figure S6.** Age standardised prevalence of Atrial fibrillation and hypertension by index of deprivation decile. **Figure S7.** Missed diagnoses of A) Hypertension and B) Atrial fibrillation by index of deprivation decile. Treatment achievement rates of A) Hypertension and B) Anti-coagulation and C)risk assessment by index of deprivation decile.

## Data Availability

Data presented in this study are freely available. Full data for this study can downloaded from https://digital.nhs.uk/data-and-information/publications/statistical/quality-and-outcomes-framework-achievement-prevalence-and-exceptions-data; https://opendata.nhsbsa.net/dataset/english-prescribing-data-epd; https://digital.nhs.uk/data-and-information/publications/statistical/patients-registered-at-a-gp-practice/september-2022; https://www.ons.gov.uk/peoplepopulationandcommunity/populationandmigration/populationestimates/datasets/clinicalcommissioninggroupmidyearpopulationestimates and English indices of deprivation 2019 - GOV.UK (www.gov.uk).

## Abbreviations

AF: Atrial Fibrillation
BP: Blood pressure
BNF: British national formulary
CVD: Cardiovascular disease
CCG: Clinical Commissioning Group
EPD: English Prescribing Dataset
GP: General practitioner
LSOA: Lower Layer Super Output Area
NHAIS: National Health Application and Infrastructure Services
NHS BSA: National Health Service Business Services Authority
NICE: National Institute of Health and Care Excellence
IMD: ONS index of multiple deprivation
PHE: Public Health England
ONS: Office for National Statistics
QOF: Quality and Outcomes Framework
sub-ICB: Sub Integrated Care Board
