# Supplementary material for: The impact of the COVID-19 pandemic on cardiovascular disease prevention and corresponding geographical inequalities in England: interrupted time series analysis
Source: BMC Public Health. 2023 Dec 7;23:2443. doi: 10.1186/s12889-023-17282-3 (PMC10702024; doi:10.1186/s12889-023-17282-3)
Supplement: Supplementary file 1 — Additional file 1: Supplemental methods. Table S1. List of paragraphs from BNF chapter 2 according to clinical indication. Table S2. Summary of data sources, calendar years and population used by analysis and disease. Table S3. Sub-ICB age standardised hypertension prevalence. Top 10 Sub-ICBs with the highest and lowest prevalence. Table S4. Sub-ICB age standardised atrial fibrillation prevalence. Top 10 Sub-ICBs with the highest and lowest prevalence. Table S5. LSOAs age standardised hypertension prevalence. Top 10 LSOAs with the highest and lowest prevalence. Table S6. LSOAs age standardised atrial fibrillation prevalence. Top 10 LSOAs with the highest and lowest prevalence. Table S7. Results of the overall ITS analysis on the Sub-IBC level. Table S8. Missed diagnoses of hypertension and impact on cardiovascular disease by Region. Table S9. Sub-ICBs hypertension treatment achievement rates. Top 10 sub-ICBs with the highest and lowest achievement rates. Table S10. Hypertension treatment achievement rate (2021-22) by Region. Table S11. LSOAs hypertension treatment achievement rates. Top 10 LSOAs with the highest and lowest achievement rate. Table S12. LSOAs Atrial Fibrillation risk assessment treatment achievement rate. Top 10 LSOAs with the highest and lowest achievement rates. Table S13. CCGs percent difference in monthly prescription rates of oral anticoagulants medication between March 2020 – April 2022. Top 10 CCGs with the highest and lowest percentage difference. Table S14. CCGs percent difference in monthly prescription rates of hypertension and heart failure medication between March 2020 – April 2022. Top 10 CCGs with the highest and lowest percentage difference. Figure S1. Observed and predicted prevalence of hypertension pre and post COVID-19 in England. Figure S2. Observed and predicted prevalence of atrial fibrillation pre and post COVID-19 in England. Figure S3. Age standardised prevalence rates of A) Hypertension Figure S3. Missed diagnoses of atrial [file 12889_2023_17282_MOESM1_ESM.docx]

**Supplemental file**

# Supplemental methods

# 1.1 Over-” and “under-estimations” of prevalence associated with assumptions made when mapping GP level data to LSOA

Where most people within two or more LSOAs attend the same GP, the reported hypertension prevalence for a single GP may be the dominant prevalence rate for multiple LSOAs, despite potentially vastly differing age distributions across the LSOAs. This could lead to “over-” and “under-estimations” of prevalence.

To investigate the magnitude of this problem in the study, we looked for all instances where at least 90% of the population for multiple LSOAs attended the same GP. Then, using median age data at GP and LSOA level, we looked for all instances where there was at least a 10-year age gap.

Among the 32,844 LSOAs in England, 218, or 0.6% of all LSOAs fulfilled the criteria, suggesting that this bias was relatively infrequent. This bias will have the biggest impact when assessing geographical differences at LSOA level. We therefore present results at Sub-ICB level in the main text and only present LSOA level data in the supplement.

# 1.2 Geographical boundary changes when moving from CCG to Sub-ICB boundaries

These changes caused 6 boundary changes and 5 NHS regions border changes.

Details of the changes are listed here:

- NHS Bassetlaw CCG has been relocated in its entirety from NHS South Yorkshire ICB to NHS Nottingham and Nottinghamshire Health ICB and relabelled Nottingham and Nottinghamshire Health ICB – 02Q. The change has moved Bassetlaw from the North-East and Yorkshire Region to the Midlands.
- A part NHS Tameside and Glossop CCG (Now NHS Greater Manchester – 01Y) has moved to NHS Derby and Derbyshire ICB – 15M
- Part of the West Birmingham area, previously in NHS Black Country and West Birmingham CCG (Now NHS Black Country ICB – D2P2L) has been moved to NHS Birmingham and Solihull ICB – 15E
- Part of Oundle has been moved from the Cambridgeshire and Peterborough CCG (Now NHS Cambridgeshire and Peterborough ICB – 06H) to NHS Northamptonshire ICB – 78H. However, the Wansford and Kings Cliffe GP was retained by NHS Cambridgeshire and Peterborough ICB – 06H.
- Border changes have reduced the size of the North-East and Yorkshire, North-West, and East of England regions, while increasing the size of the South-East and Midlands.

# 1.3 Age standardisation

Prevalence estimates have been age-sex adjusted using an indirect standardisation approach. For hypertension, LSOA age structure was used to inform the GP age profile. Age and sex specific hypertension prevalence estimates from the 2019 Health Survey for England^1^ were applied to 2022 age and sex stratified population estimates data from the NHAIS System.^2^ For atrial fibrillation, CCG age structure was used to inform the GP age profile. Age and sex specific AF prevalence estimates from CVD prevent^3^ were applied to 2022 age and sex stratified population estimates data from the NHAIS System.^2^

$$ExpectedPrev=Age_{1}AgeExpPrev_{1}+Age_{2}AgeExpPrev_{2}+\ldots+Age_{n}AgeExpPrev_{n}$$

where 1, 2, …, n are the age/sex groups and expected prevalence for those age/sex groups.

The expected prevalence rate of hypertension/AF is then used alongside the observed prevalence to indirectly standardise the rate of hypertension/AF at GP level using the following formula:

$$AgeStdPrev_{i}= \frac{ObservedPrev_{i}}{ExpectedPrev_{i}} \times ObservedPrev$$

Where $i=1, 2, \ldots, n$ for all *n* GPs in England. ObservedPrev refers to the national overall prevalence rate.

# 1.4 Estimation of additional CVD events

Dale et al.^4^ estimated one CVD event (including stable and unstable angina, stroke, myocardial infraction (MI), transient ischemic heart attack and heart failure) occurs for each 35.96 cases of hypertension that go undiagnosed over a lifetime. The figure for stroke is one in 141.4 and for MI it is one in 215.4. If one were to diagnose and treat these cases within 5 years the ratio decreases to one in 180.9 CVD events, one in 1,068 strokes and one in 675.5 MIs.

# References

1. NHS Digital. Health Survey for England 2019: Adults’ health. Prevalence of hypertension, by survey year, age and sex, Table 9. [Internet]. NHS Digital. [cited 2022 Nov 3]. Available from: https://digital.nhs.uk/data-and-information/publications/statistical/health-survey-for-england

2. NHS Digital. Patients Registered at a GP Practice, September 2022 [Internet]. NHS Digital. 2022 [cited 2022 Nov 7]. Available from: https://digital.nhs.uk/data-and-information/publications/statistical/patients-registered-at-a-gp-practice/september-2022

3. Office for Health Improvement and Disparities and the NHS Benchmarking Network. Cardiovascular Disease Prevention Audit (CVDPREVENT). [Internet]. CVDPREVENT. 2021 [cited 2022 Nov 3]. Available from: https://www.cvdprevent.nhs.uk/data-extract?indicator=11&systemLevel=3&period=4

4. Dale CE, Takhar R, Carragher R, Katsoulis M, Torabi F, Duffield S, et al. The impact of the COVID-19 pandemic on cardiovascular disease prevention and management. Nat Med. 2023 Jan;29(1):219–25.

# Tables

## Table S1. List of paragraphs from BNF chapter 2 according to clinical indication

| **BNF subsection** | **BNF code** | **BNF title** | **Clinical indication** |
| --- | --- | --- | --- |
| *Section* | *2.1* | *Positive inotropic drugs* |  |
| Subsection | 2.1.1 | Cardiac Glycosides | Exclude |
| Subsection | 2.1.2 | Phosphodiesterase Type-3 inhibitors | Exclude |
| *Section* | *2.2* | *Diuretics* |  |
| Subsection | 2.2.1 | Thiazides and related diuretics | Hypertension |
| Subsection | 2.2.2 | Loop diuretics | Both (HTN & HF) |
| Subsection | 2.2.3 | Potassium-sparing diuretics and aldosterone antagonists | Both (HTN & HF) |
| Subsection | 2.2.4 | Potassium sparing diuretics and compounds | Both (HTN & HF) |
| Subsection | 2.2.5 | Osmotic diuretics | Exclude |
| Subsection | 2.2.8 | Diuretics with potassium | Both (HTN & HF) |
| *Section* | *2.3* | *Anti-arrhythmic drugs* |  |
| Subsection | 2.3.2 | Drugs for arrhythmias | Exclude |
| Section | 2.4 | Beta-adrenoceptor blocking drugs | Both (HTN & HF) |
| Section | 2.5 | Hypertension and heart failure |  |
| Subsection | 2.5.1 | Vasodilator antihypertensive drugs | Exclude |
| Subsection | 2.5.2 | Centrally-acting antihypertensive drugs | Hypertension |
| Subsection | 2.5.3 | Adrenergic neurone blocking drugs | Exclude |
| Subsection | 2.5.4 | Alpha-adrenoceptor blocking drugs | Hypertension |
| Subsection | 2.5.5 | Renin-angiotensin system drugs | Both (HTN & HF) |
| Subsection | 2.5.8 | Other adrenergic neurone blocking drugs | Hypertension |
| *Section* | *2.6* | *Nitrates, calcium-channel blockers & other antianginal drugs* | |
| Subsection | 2.6.1 | Nitrates | Heart Failure |
| Subsection | 2.6.2 | Calcium-channel blockers | Hypertension |
| Subsection | 2.6.3 | Other antianginal drugs | Exclude |
| Subsection | 2.6.4 | Peripheral vasodilators and related drugs | Exclude |
| *Section* | *2.7* | *Sympathomimetics* |  |
| Subsection | 2.7.1 | Inotropic sympathomimetics | Exclude |
| Subsection | 2.7.2 | Vasoconstrictor sympathomimetics | Exclude |
| Subsection | 2.7.3 | Cardiopulmonary resuscitation | Exclude |
| *Section* | *2.8* | *Anticoagulants and protamine* |  |
| Subsection | 2.8.1 | Parental anticoagulants | Exclude |
| Subsection | 2.8.2 | Oral anticoagulants | Anticoagulants |
| Subsection | 2.8.3 | Protamine sulphate | Exclude |
| *Section* | *2.9* | *Antiplatelet drugs* | *Exclude* |
| *Section* | *2.1* | *Stable angina, acute coronary syndromes and fibrinolysis* | *Exclude* |
| Subsection | 2.10.2 | Fibrinolytic drugs | Exclude |
| *Section* | *2.11* | *Antifibrinolytic drugs and haemostatics* | *Exclude* |
| *Section* | *2.12* | *Lipid-regulating drugs* | *Exclude* |
| *Section* | *2.13* | *Local sclerosants* | *Exclude* |

*HTN: hypertension and HF: heart failure

## Table S2. Summary of data sources, calendar years and population used by analysis and disease

| Analysis | **Data Source** | **Years** | **Total population (2021/22)** |
| --- | --- | --- | --- |
| **Hypertension** |  |  |  |
| Prevalence and treatment indicators | Quality and Outcomes Framework (QoF) | April 2016 to March 2022 | 61,727,641^1^ |
| **Atrial Fibrillation** |  |  |  |
| Prevalence and treatment indicators | Quality and Outcomes Framework (QoF) | April 2016 to March 2022 | 61,699,624^1^ |
| **Prescription** |  |  |  |
| Number of items | English Prescribing Dataset (EPD) | Apr 2018 – Apr 2022 | 56,550,138^2^ |

^1^ NHS Digital. Patients Registered at a GP Practice, September 2022 [Internet]. NHS Digital. 2022 [cited 2022 Nov 7]. Available from: <https://digital.nhs.uk/data-and-information/publications/statistical/patients-registered-at-a-gp-practice/september-2022>. Note: populations differ between the hypertension and the AF analysis because not all GP practices report metrics for both diseases.

^2^ Sum of all CCG populations of mid-year estimates 2020 from Clinical commissioning group population estimates (National Statistics) - Office for National Statistics [Internet]. [cited 2023 Mar 27]. Available from: https://www.ons.gov.uk/peoplepopulationandcommunity/populationandmigration/populationestimates/datasets/clinicalcommissioninggroupmidyearpopulationestimates

## Table S3. Sub-ICB age standardised hypertension prevalence. Top 10 Sub-ICBs with the highest and lowest prevalence

| **10 Highest Hypertension Prevalence Sub-ICB Locations (2021/22)** | | | |  | **10 Lowest Hypertension Prevalence Sub-ICB Locations (2021/22)** | | |
| --- | --- | --- | --- | --- | --- | --- | --- |
| **Sub-ICB Location** | **Age Standardised Hypertension Prevalence** | | **Region** |  | **Sub-ICB Location** | **Age Standardised Hypertension Prevalence** | **Region** |
| NHS Staffordshire and Stoke-on-Trent ICB - 05W | | 17.71% | Midlands |  | NHS Sussex ICB - 09D | 12.17% | South East |
| NHS North East and North Cumbria ICB - 00P | | 17.19% | North East and Yorkshire |  | NHS Surrey Heartlands ICB - 92A | 13.45% | South East |
| NHS Lancashire and South Cumbria ICB - 00R | | 17.16% | North West |  | NHS Humber and North Yorkshire ICB - 03Q | 13.74% | North East and Yorkshire |
| NHS Mid and South Essex ICB - 07G | | 17.16% | East of England |  | NHS South West London ICB - 36L | 13.87% | London |
| NHS Greater Manchester ICB - 01Y | | 17.04% | North West |  | NHS Dorset ICB - 11J | 13.89% | South West |
| NHS Leicester, Leicestershire and Rutland ICB - 04C | | 16.99% | Midlands |  | NHS Cornwall and the Isles of Scilly ICB - 11N | 13.93% | South West |
| NHS Cheshire and Merseyside ICB - 01F | | 16.92% | North West |  | NHS Gloucestershire ICB - 11M | 13.94% | South West |
| NHS Cheshire and Merseyside ICB - 01X | | 16.90% | North West |  | NHS Hertfordshire and West Essex ICB - 06N | 14.03% | East of England |
| NHS Cheshire and Merseyside ICB - 01J | | 16.90% | North West |  | NHS Buckinghamshire, Oxfordshire and Berkshire West ICB - 10Q | 14.04% | South East |
| NHS Black Country ICB - D2P2L | | 16.82% | Midlands |  | NHS Lancashire and South Cumbria ICB - 01K | 14.13% | North West |

## Table S4. Sub-ICB age standardised atrial fibrillation prevalence. Top 10 Sub-ICBs with the highest and lowest prevalence

| **Highest AF Prevalence Sub-ICB Locations (2021/22)** | | |  | **Lowest AF Prevalence Sub-ICB Locations (2021/22)** | | |
| --- | --- | --- | --- | --- | --- | --- |
| **Sub-ICB Location** | **Age Standardised AF Prevalence** | **Region** |  | **Sub-ICB Location** | **Age Standardised AF Prevalence** | **Region** |
| NHS Cheshire and Merseyside ICB - 12F | 3.32% | North West |  | NHS North East London ICB - A3A8R | 1.91% | London |
| NHS Cheshire and Merseyside ICB - 01F | 3.29% | North West |  | NHS Leicester, Leicestershire and Rutland ICB - 04C | 1.98% | Midlands |
| NHS Nottingham and Nottinghamshire ICB - 02Q | 3.13% | Midlands |  | NHS North West London ICB - W2U3Z | 2.07% | London |
| NHS Cheshire and Merseyside ICB - 01X | 3.10% | North West |  | NHS North Central London ICB - 93C | 2.11% | London |
| NHS Sussex ICB - 97R | 3.10% | South East |  | NHS South East London ICB - 72Q | 2.14% | London |
| NHS Humber and North Yorkshire ICB - 03H | 3.07% | North East and Yorkshire |  | NHS South West London ICB - 36L | 2.23% | London |
| NHS Cheshire and Merseyside ICB - 01V | 3.05% | North West |  | NHS Birmingham and Solihull ICB - 15E | 2.27% | Midlands |
| NHS Cheshire and Merseyside ICB - 27D | 3.03% | North West |  | NHS Greater Manchester ICB - 14L | 2.36% | North West |
| NHS Cheshire and Merseyside ICB - 01T | 3.01% | North West |  | NHS West Yorkshire ICB - 36J | 2.41% | North East and Yorkshire |
| NHS North East and North Cumbria ICB - 00P | 3.00% | North East and Yorkshire |  | NHS West Yorkshire ICB - X2C4Y | 2.47% | North East and Yorkshire |

AF = Atrial Fibrillation

## Table S5. LSOAs age standardised hypertension prevalence. Top 10 LSOAs with the highest and lowest prevalence

| **Highest Hypertension Prevalence LSOAs (2021/22)** | | |  | **Lowest Hypertension Prevalence LSOAs (2021/22)** | | |
| --- | --- | --- | --- | --- | --- | --- |
| **LSOA** | **Age Standardised Hypertension Prevalence** | **Sub-ICB Location** |  | **LSOA** | **Age Standardised Hypertension Prevalence** | **Sub-ICB Location** |
| Ealing 023A | 22.13% | NHS North West London ICB - W2U3Z |  | Cornwall 035D | 6.94% | NHS Cornwall and the Isles of Scilly ICB - 11N |
| Ealing 026E | 22.10% | NHS North West London ICB - W2U3Z |  | Cornwall 035B | 7.81% | NHS Cornwall and the Isles of Scilly ICB - 11N |
| Ealing 023C | 21.94% | NHS North West London ICB - W2U3Z |  | Brighton and Hove 002B | 8.33% | NHS Sussex ICB - 09D |
| Ealing 017C | 21.84% | NHS North West London ICB - W2U3Z |  | Kensington and Chelsea 012A | 8.51% | NHS North West London ICB - W2U3Z |
| Ealing 026B | 21.82% | NHS North West London ICB - W2U3Z |  | Richmond upon Thames 022F | 8.56% | NHS South West London ICB - 36L |
| Ealing 026F | 21.81% | NHS North West London ICB - W2U3Z |  | Richmond upon Thames 022A | 8.59% | NHS South West London ICB - 36L |
| Ealing 026D | 21.79% | NHS North West London ICB - W2U3Z |  | Kensington and Chelsea 012B | 8.65% | NHS North West London ICB - W2U3Z |
| Ealing 023E | 21.79% | NHS North West London ICB - W2U3Z |  | Kensington and Chelsea 012D | 8.66% | NHS North West London ICB - W2U3Z |
| Ealing 017D | 21.46% | NHS North West London ICB - W2U3Z |  | Westminster 019C | 8.69% | NHS North West London ICB - W2U3Z |
| Haringey 006B | 21.42% | NHS North Central London ICB - 93C |  | Kensington and Chelsea 019E | 8.72% | NHS North West London ICB - W2U3Z |
| Ealing 026A | 21.39% | NHS North West London ICB - W2U3Z |  | Kensington and Chelsea 018B | 8.73% | NHS North West London ICB - W2U3Z |
| Ealing 017E | 21.34% | NHS North West London ICB - W2U3Z |  | Kensington and Chelsea 012E | 8.77% | NHS North West London ICB - W2U3Z |
| Ealing 017B | 21.31% | NHS North West London ICB - W2U3Z |  | Kensington and Chelsea 014E | 8.78% | NHS North West London ICB - W2U3Z |
| Ealing 026C | 21.30% | NHS North West London ICB - W2U3Z |  | Kensington and Chelsea 014D | 8.81% | NHS North West London ICB - W2U3Z |
| Haringey 037D | 21.21% | NHS North Central London ICB - 93C |  | Kensington and Chelsea 018E | 8.82% | NHS North West London ICB - W2U3Z |
| Ealing 037B | 21.20% | NHS North West London ICB - W2U3Z |  | Kensington and Chelsea 018A | 8.82% | NHS North West London ICB - W2U3Z |
| Ealing 037C | 21.16% | NHS North West London ICB - W2U3Z |  | Cornwall 028D | 8.86% | NHS Cornwall and the Isles of Scilly ICB - 11N |
| County Durham 020A | 21.13% | NHS North East and North Cumbria ICB - 84H |  | Kensington and Chelsea 014A | 8.90% | NHS North West London ICB - W2U3Z |
| Ealing 037A | 21.03% | NHS North West London ICB - W2U3Z |  | Richmond upon Thames 022D | 8.90% | NHS South West London ICB - 36L |
| Ealing 037D | 21.02% | NHS North West London ICB - W2U3Z |  | Kensington and Chelsea 010B | 8.91% | NHS North West London ICB - W2U3Z |
| Ealing 014E | 20.98% | NHS North West London ICB - W2U3Z |  | Kensington and Chelsea 019A | 8.93% | NHS North West London ICB - W2U3Z |
| Ealing 017A | 20.97% | NHS North West London ICB - W2U3Z |  | Westminster 019F | 9.00% | NHS North West London ICB - W2U3Z |
| Newham 011A | 20.94% | NHS North East London ICB - A3A8R |  | Kensington and Chelsea 014C | 9.03% | NHS North West London ICB - W2U3Z |
| Bolsover 007B | 20.92% | NHS Derby and Derbyshire ICB - 15M |  | Kensington and Chelsea 012C | 9.03% | NHS North West London ICB - W2U3Z |
| Ealing 037E | 20.86% | NHS North West London ICB - W2U3Z |  | Kensington and Chelsea 010E | 9.04% | NHS North West London ICB - W2U3Z |

LSOA = Lower Super Output Area

## Table S6. LSOAs age standardised atrial fibrillation prevalence. Top 10 LSOAs with the highest and lowest prevalence

| **Highest AF Prevalence LSOAs (2021/22)** | | |  | **Lowest AF Prevalence LSOAs (2021/22)** | | |
| --- | --- | --- | --- | --- | --- | --- |
| **LSOA** | **Age Standardised AF Prevalence** | **Sub-ICB Location** |  | **LSOA** | **Age Standardised AF Prevalence** | **Sub-ICB Location** |
| Kensington and Chelsea 018C | 4.15% | NHS North West London ICB - W2U3Z |  | Leicester 010E | 1.10% | NHS Leicester, Leicestershire and Rutland ICB - 04C |
| Northumberland 019C | 3.77% | NHS North East and North Cumbria ICB - 00L |  | Leicester 010A | 1.11% | NHS Leicester, Leicestershire and Rutland ICB - 04C |
| Ashford 012C | 3.74% | NHS Kent and Medway ICB - 91Q |  | Leicester 006C | 1.13% | NHS Leicester, Leicestershire and Rutland ICB - 04C |
| Wirral 025B | 3.67% | NHS Cheshire and Merseyside ICB - 12F |  | Newham 008D | 1.14% | NHS North East London ICB - A3A8R |
| Swindon 016A | 3.65% | NHS Bath and North East Somerset, Swindon and Wiltshire ICB - 92G |  | Leicester 010B | 1.14% | NHS Leicester, Leicestershire and Rutland ICB - 04C |
| Wigan 004D | 3.65% | NHS Greater Manchester ICB - 02H |  | Leicester 007B | 1.15% | NHS Leicester, Leicestershire and Rutland ICB - 04C |
| Wirral 025A | 3.63% | NHS Cheshire and Merseyside ICB - 12F |  | Newham 008E | 1.16% | NHS North East London ICB - A3A8R |
| Wigan 004A | 3.62% | NHS Greater Manchester ICB - 02H |  | Leicester 006D | 1.16% | NHS Leicester, Leicestershire and Rutland ICB - 04C |
| Rother 002A | 3.61% | NHS Sussex ICB - 97R |  | Leicester 007E | 1.17% | NHS Leicester, Leicestershire and Rutland ICB - 04C |
| Wigan 004C | 3.61% | NHS Greater Manchester ICB - 02H |  | Brent 029E | 1.18% | NHS North West London ICB - W2U3Z |
| Rother 004C | 3.58% | NHS Sussex ICB - 97R |  | Leicester 022A | 1.18% | NHS Leicester, Leicestershire and Rutland ICB - 04C |
| Wealden 018B | 3.58% | NHS Sussex ICB - 97R |  | Leicester 010D | 1.18% | NHS Leicester, Leicestershire and Rutland ICB - 04C |
| Wirral 025C | 3.56% | NHS Cheshire and Merseyside ICB - 12F |  | Leicester 006A | 1.19% | NHS Leicester, Leicestershire and Rutland ICB - 04C |
| Rother 004B | 3.56% | NHS Sussex ICB - 97R |  | Leicester 018B | 1.19% | NHS Leicester, Leicestershire and Rutland ICB - 04C |
| Halton 003E | 3.55% | NHS Cheshire and Merseyside ICB - 01F |  | Brent 019C | 1.19% | NHS North West London ICB - W2U3Z |
| Northumberland 019A | 3.55% | NHS North East and North Cumbria ICB - 00L |  | Leicester 022B | 1.19% | NHS Leicester, Leicestershire and Rutland ICB - 04C |
| Halton 007B | 3.55% | NHS Cheshire and Merseyside ICB - 01F |  | Brent 026C | 1.20% | NHS North West London ICB - W2U3Z |
| Teignbridge 017B | 3.55% | NHS Devon ICB - 15N |  | Brent 019D | 1.20% | NHS North West London ICB - W2U3Z |
| Bedford 001C | 3.55% | NHS Bedfordshire, Luton and Milton Keynes ICB - M1J4Y |  | Brent 029B | 1.20% | NHS North West London ICB - W2U3Z |
| Wirral 025E | 3.55% | NHS Cheshire and Merseyside ICB - 12F |  | Newham 008C | 1.21% | NHS North East London ICB - A3A8R |
| Halton 001D | 3.54% | NHS Cheshire and Merseyside ICB - 01F |  | Brent 026B | 1.21% | NHS North West London ICB - W2U3Z |
| Teignbridge 017A | 3.54% | NHS Devon ICB - 15N |  | Leicester 007C | 1.21% | NHS Leicester, Leicestershire and Rutland ICB - 04C |
| St. Helens 001B | 3.54% | NHS Cheshire and Merseyside ICB - 01X |  | Newham 017C | 1.21% | NHS North East London ICB - A3A8R |
| Cheshire West and Chester 035C | 3.54% | NHS Cheshire and Merseyside ICB - 27D |  | Birmingham 040A | 1.23% | NHS Birmingham and Solihull ICB - 15E |
| Halton 003A | 3.54% | NHS Cheshire and Merseyside ICB - 01F |  | Birmingham 051E | 1.23% | NHS Birmingham and Solihull ICB - 15E |

LSOA = Lower Super Output Area
AF = Atrial Fibrillation

## Table S7. Results of the overall ITS analysis on the Sub-IBC level

| **Term*** | **Estimate** | **Std. Error** | **T-Statistic** | **p-value** |
| --- | --- | --- | --- | --- |
| **Hypertension ITS coefficients** |  |  |  |  |
| Intercept | 14.542 | 0.162 | 90.02 | <0.0001 |
| year | 0.102 | 0.011 | 9.31 | <0.0001 |
| COVID | -0.54 | 0.166 | -3.258 | 0.127 |
| year:COVID | 0.047 | 0.026 | 1.824 | 0.07 |
| **AF ITS coefficients** |  |  |  |  |
| Intercept | 1.808 | 0.04 | 45.667 | <0.0001 |
| year | 0.103 | 0.003 | 30.68 | <0.0001 |
| COVID | 0.073 | 0.038 | 1.912 | 0.056 |
| year:COVID | -0.036 | 0.007 | -5.141 | 0.001 |
| **Prescription ITS coefficients - Heart Failure Medications** | | |  |  |
| Intercept | 0.361 | 0.01 | 34.443 | <0.0001 |
| Time in months | 0.000 | <0.001 | -7.083 | <0.0001 |
| COVID* | -0.008 | 0.001 | -5.757 | <0.0001 |
| Time in months:COVID | 0.000 | <0.001 | -0.207 | 0.847 |
| **Prescription ITS coefficients - Hypertension and Heart Failure Medications** | | | |  |
| Intercept | 6.26 | 0.115 | 54.496 | <0.0001 |
| Time in months | 0.008 | 0.001 | 10.217 | <0.0001 |
| COVID | 0.047 | 0.021 | 2.238 | 0.025 |
| Time in months:COVID | -0.004 | 0.001 | -5.285 | <0.0001 |
| **Prescription ITS coefficients - Hypertension Medications** | | |  |  |
| Intercept | 3.212 | 0.042 | 76.301 | <0.0001 |
| Time in months | 0.003 | <0.001 | 7.5 | <0.0001 |
| COVID | 0.035 | 0.011 | 3.23 | 0.001 |
| Time in months:COVID | -0.002 | <0.001 | -5.181 | <0.0001 |
| **Prescription ITS coefficients - Oral Anticoagulants** | | |  |  |
| Intercept | 0.887 | 0.021 | 42.356 | <0.0001 |
| Time in months | 0.004 | <0.001 | 21.696 | <0.0001 |
| COVID | 0.041 | 0.004 | 11.752 | <0.0001 |
| Time in months:COVID | -0.002 | <0.001 | -15.646 | <0.0001 |
| **Prescription ITS coefficients - Total of medication classes studied** | | | |  |
| Intercept | 10.719 | 0.189 | 56.835 | <0.0001 |
| Time in months | 0.015 | 0.001 | 10.852 | <0.0001 |
| COVID | 0.116 | 0.036 | 3.19 | 0.002 |
| Time in months:COVID | -0.009 | 0.001 | -6.162 | <0.0001 |
| *The formula in the methods expresses the intercept as beta_0;_ year/ Time in months as beta_1_T; COVID as beta_2_; and year/ Time in months:COVID as beta_3_ | | | | |

## Table S8. Missed diagnoses of hypertension and impact on cardiovascular disease by Region.

| **Region** | **Crude Hypertension Prevalence** | **Regional Population** | **Difference from expected*** | **Difference per 100,000*** | **95% CI*** | **Preventable Lifetime Untreated events** | | | **Preventable Treated events Within 5 Years** | | |
| --- | --- | --- | --- | --- | --- | --- | --- | --- | --- | --- | --- |
|  |  |  |  |  |  | **CVD Events** | **Strokes** | **MI** | **CVD Events** | **Strokes** | **MI** |
| East of England | 14.39% | 7,118,404 | -16,882 | -237 | (-924, 450) | 469 | 119 | 78 | 93 | 16 | 25 |
| London | 11.40% | 10,636,722 | 2,669 | 25 | (-284, 334) | 0 | 0 | 0 | 0 | 0 | 0 |
| Midlands | 14.89% | 11,617,691 | -38,299 | -330 | (-883, 224) | 1065 | 271 | 178 | 212 | 36 | 57 |
| North East and Yorkshire | 15.32% | 9,021,124 | -23,167 | -257 | (-740, 226) | 644 | 164 | 108 | 128 | 22 | 34 |
| North West | 14.67% | 7,695,397 | -27,650 | -359 | (-972, -253) | 769 | 196 | 128 | 153 | 26 | 41 |
| South East | 14.23% | 9,628,611 | -33,391 | -347 | (-1,443, 750) | 929 | 236 | 155 | 185 | 31 | 49 |
| South West | 15.20% | 6,009,692 | -7,102 | -118 | (-1,131,894) | 197 | 50 | 33 | 39 | 7 | 11 |

*These results were obtained from the interrupted time series analysis.

## Table S9. Sub-ICBs hypertension treatment achievement rates. Top 10 sub-ICBs with the highest and lowest achievement rates.

| **Highest Treatment Sub-ICB Locations (2021/22)** | | | |  | **Lowest Treatment Sub-ICB Locations (2021/22)** | | | |
| --- | --- | --- | --- | --- | --- | --- | --- | --- |
| **Sub-ICB Location** | **Hypertension Treatment Rate** | **Treated Population** | **Region** |  | **Sub-ICB Location** | **Hypertension Treatment Rate** | **Treated Population** | **Region** |
| NHS Humber and North Yorkshire ICB - 42D | 74.78% | 56,444 | North East and Yorkshire |  | NHS Greater Manchester ICB - 00V | 56.21% | 16,101 | North West |
| NHS Mid and South Essex ICB - 07G | 73.82% | 19,126 | East of England |  | NHS Cheshire and Merseyside ICB - 01J | 56.56% | 14,760 | North West |
| NHS North East and North Cumbria ICB - 16C | 72.63% | 81,704 | North East and Yorkshire |  | NHS Mid and South Essex ICB - 99F | 56.84% | 18,528 | East of England |
| NHS North East and North Cumbria ICB - 84H | 71.60% | 68,942 | North East and Yorkshire |  | NHS Staffordshire and Stoke-on-Trent ICB - 05V | 56.98% | 14,135 | Midlands |
| NHS Leicester, Leicestershire and Rutland ICB - 04C | 71.10% | 37,082 | Midlands |  | NHS Greater Manchester ICB - 02A | 58.74% | 20,179 | North West |
| NHS North East and North Cumbria ICB - 01H | 70.93% | 38,923 | North East and Yorkshire |  | NHS Black Country ICB - D2P2L | 58.78% | 116,009 | Midlands |
| NHS Cambridgeshire and Peterborough ICB - 06H | 70.82% | 91,046 | East of England |  | NHS Kent and Medway ICB - 91Q | 59.26% | 172,894 | South East |
| NHS South Yorkshire ICB - 02X | 70.78% | 35,876 | North East and Yorkshire |  | NHS Sussex ICB - 97R | 59.66% | 57,812 | South East |
| NHS Nottingham and Nottinghamshire ICB - 02Q | 70.53% | 13,111 | Midlands |  | NHS Sussex ICB - 09D | 59.79% | 18,519 | South East |
| NHS Suffolk and North East Essex ICB - 06L | 70.50% | 46,607 | East of England |  | NHS Cheshire and Merseyside ICB - 01T | 59.97% | 15,337 | North West |

## Table S10. Hypertension treatment achievement rate (2021-22) by Region.

| **Region** | **Treatment Rate in 2021/22** |
| --- | --- |
| East of England | 65.94% |
| London | 63.09% |
| Midlands | 64.68% |
| North-East and Yorkshire | 68.82% |
| North West | 64.13% |
| South East | 61.08% |
| South West | 65.38% |

## Table S11. LSOAs hypertension treatment achievement rates. Top 10 LSOAs with the highest and lowest achievement rate.

| ***Lowest Treatment Rate LSOAs*** | | | |
| --- | --- | --- | --- |
| **LSOA Name** | **Observed Hypertension Prevalence** | **Treatment Rate** | **Sub-ICB Name** |
| Tonbridge and Malling 006D | 15.16 | 27.32 | NHS Kent and Medway ICB - 91Q |
| Tonbridge and Malling 006C | 15.16 | 27.36 | NHS Kent and Medway ICB - 91Q |
| Tonbridge and Malling 006B | 15.16 | 27.42 | NHS Kent and Medway ICB - 91Q |
| Tonbridge and Malling 006F | 15.06 | 28.66 | NHS Kent and Medway ICB - 91Q |
| Tonbridge and Malling 006E | 15.09 | 29.01 | NHS Kent and Medway ICB - 91Q |
| Birmingham 015D | 14.51 | 36.56 | NHS Birmingham and Solihull ICB - 15E |
| Shepway 009D | 16.59 | 36.90 | NHS Kent and Medway ICB - 91Q |
| Stafford 015C | 16.50 | 37.09 | NHS Staffordshire and Stoke-on-Trent ICB - 05V |
| Stafford 015E | 16.50 | 37.11 | NHS Staffordshire and Stoke-on-Trent ICB - 05V |
| Gravesham 013C | 14.05 | 38.06 | NHS Kent and Medway ICB - 91Q |
| Milton Keynes 005C | 13.24 | 38.12 | NHS Bedfordshire, Luton and Milton Keynes ICB - M1J4Y |
| Haringey 004D | 9.48 | 38.14 | NHS North Central London ICB - 93C |
| Bexley 028C | 14.50 | 38.29 | NHS South East London ICB - 72Q |
| Bexley 028D | 14.37 | 38.33 | NHS South East London ICB - 72Q |
| Shepway 003B | 13.36 | 38.62 | NHS Kent and Medway ICB - 91Q |
| Birmingham 015B | 14.25 | 38.73 | NHS Birmingham and Solihull ICB - 15E |
| Aylesbury Vale 022F | 14.60 | 38.79 | NHS Buckinghamshire, Oxfordshire and Berkshire West ICB - 14Y |
| Aylesbury Vale 022A | 14.55 | 38.80 | NHS Buckinghamshire, Oxfordshire and Berkshire West ICB - 14Y |
| Sandwell 006C | 15.64 | 38.82 | NHS Black Country ICB - D2P2L |
| Sevenoaks 016C | 16.94 | 38.84 | NHS Kent and Medway ICB - 91Q |
| Mid Suffolk 001A | 15.73 | 38.91 | NHS Suffolk and North East Essex ICB - 06L |
| Sevenoaks 016B | 16.88 | 38.94 | NHS Kent and Medway ICB - 91Q |
| Aylesbury Vale 022C | 14.57 | 38.98 | NHS Buckinghamshire, Oxfordshire and Berkshire West ICB - 14Y |
| Wealden 020B | 15.59 | 39.03 | NHS Sussex ICB - 97R |
| Bexley 028B | 14.24 | 39.07 | NHS South East London ICB - 72Q |
|  |  |  |  |
| ***Highest Treatment Rate LSOAs*** | | | |
| **LSOA Name** | **Observed Hypertension Prevalence** | **Treatment Rate** | **Sub-ICB Name** |
| Peterborough 021C | 12.33 | 93.39 | NHS Cambridgeshire and Peterborough ICB - 06H |
| Peterborough 011D | 13.09 | 91.94 | NHS Cambridgeshire and Peterborough ICB - 06H |
| Peterborough 011A | 13.13 | 90.72 | NHS Cambridgeshire and Peterborough ICB - 06H |
| Peterborough 011E | 13.02 | 90.69 | NHS Cambridgeshire and Peterborough ICB - 06H |
| Peterborough 021B | 12.13 | 90.52 | NHS Cambridgeshire and Peterborough ICB - 06H |
| Peterborough 011B | 12.86 | 89.99 | NHS Cambridgeshire and Peterborough ICB - 06H |
| Peterborough 011C | 13.3 | 89.39 | NHS Cambridgeshire and Peterborough ICB - 06H |
| Tower Hamlets 018A | 4.56 | 89.15 | NHS North East London ICB - A3A8R |
| King's Lynn and West Norfolk 004A | 19.56 | 89.01 | NHS Norfolk and Waveney ICB - 26A |
| Peterborough 009B | 12.75 | 88.8 | NHS Cambridgeshire and Peterborough ICB - 06H |
| King's Lynn and West Norfolk 001C | 19.6 | 88.79 | NHS Norfolk and Waveney ICB - 26A |
| Peterborough 021D | 12.4 | 88.74 | NHS Cambridgeshire and Peterborough ICB - 06H |
| King's Lynn and West Norfolk 001D | 19.58 | 88.73 | NHS Norfolk and Waveney ICB - 26A |
| King's Lynn and West Norfolk 004B | 19.57 | 88.69 | NHS Norfolk and Waveney ICB - 26A |
| King's Lynn and West Norfolk 004C | 19.59 | 88.62 | NHS Norfolk and Waveney ICB - 26A |
| Peterborough 015A | 13.09 | 88.5 | NHS Cambridgeshire and Peterborough ICB - 06H |
| King's Lynn and West Norfolk 001A | 19.6 | 88.46 | NHS Norfolk and Waveney ICB - 26A |
| Peterborough 009A | 12.69 | 88.45 | NHS Cambridgeshire and Peterborough ICB - 06H |
| Peterborough 021E | 12.45 | 88.38 | NHS Cambridgeshire and Peterborough ICB - 06H |
| Tower Hamlets 020A | 4.78 | 88.25 | NHS North East London ICB - A3A8R |
| Peterborough 023A | 12.59 | 88.14 | NHS Cambridgeshire and Peterborough ICB - 06H |
| Peterborough 022A | 12.27 | 87.87 | NHS Cambridgeshire and Peterborough ICB - 06H |
| Wyre Forest 014A | 20.37 | 87.4 | NHS Herefordshire and Worcestershire ICB - 18C |
| Knowsley 006A | 14.34 | 87.34 | NHS Cheshire and Merseyside ICB - 01J |
| Wyre Forest 014B | 20.4 | 87.31 | NHS Herefordshire and Worcestershire ICB - 18C |

LSOA = Lower Super Output Area

## Table S12. LSOAs Atrial Fibrillation risk assessment treatment achievement rate. Top 10 LSOAs with the highest and lowest achievement rates.

| ***Lowest Risk Assessment Rate LSOAs*** | | |
| --- | --- | --- |
| **LSOA Name** | **Risk Assessment Rate** | **Sub-ICB** |
| North West Leicestershire 012D | 8.76 | NHS Leicester, Leicestershire and Rutland ICB - 04V |
| North West Leicestershire 012B | 9.00 | NHS Leicester, Leicestershire and Rutland ICB - 04V |
| North West Leicestershire 012C | 9.21 | NHS Leicester, Leicestershire and Rutland ICB - 04V |
| East Hertfordshire 001D | 9.65 | NHS Hertfordshire and West Essex ICB - 06K |
| East Hertfordshire 001B | 10.32 | NHS Hertfordshire and West Essex ICB - 06K |
| East Hertfordshire 001C | 13.27 | NHS Hertfordshire and West Essex ICB - 06K |
| East Hertfordshire 002B | 15.33 | NHS Hertfordshire and West Essex ICB - 06K |
| Bedford 004B | 16.88 | NHS Bedfordshire, Luton and Milton Keynes ICB - M1J4Y |
| North West Leicestershire 012A | 17.51 | NHS Leicester, Leicestershire and Rutland ICB - 04V |
| Fenland 004D | 19.67 | NHS Cambridgeshire and Peterborough ICB - 06H |
| Kirklees 042D | 19.74 | NHS West Yorkshire ICB - X2C4Y |
| Stroud 003C | 20.61 | NHS Gloucestershire ICB - 11M |
| North West Leicestershire 008D | 21.87 | NHS Leicester, Leicestershire and Rutland ICB - 04V |
| Warrington 021G | 22.04 | NHS Cheshire and Merseyside ICB - 02E |
| Rochford 007C | 22.19 | NHS Mid and South Essex ICB - 99F |
| North West Leicestershire 008E | 22.55 | NHS Leicester, Leicestershire and Rutland ICB - 04V |
| Leeds 111B | 22.62 | NHS West Yorkshire ICB - 15F |
| Rochford 004D | 23.79 | NHS Mid and South Essex ICB - 99F |
| Rochford 007E | 24.23 | NHS Mid and South Essex ICB - 99F |
| Rochford 004C | 24.40 | NHS Mid and South Essex ICB - 99F |
| Norwich 010C | 24.63 | NHS Norfolk and Waveney ICB - 26A |
| Leeds 111A | 24.75 | NHS West Yorkshire ICB - 15F |
| Warrington 021D | 24.83 | NHS Cheshire and Merseyside ICB - 02E |
| Warrington 021E | 25.09 | NHS Cheshire and Merseyside ICB - 02E |
| Milton Keynes 019C | 25.13 | NHS Bedfordshire, Luton and Milton Keynes ICB - M1J4Y |
|  |  |  |
| ***Highest Risk Assessment Rate LSOAs*** | | |
| **LSOA Name** | **Risk Assessment Rate** | **Sub-ICB Name** |
| Shropshire 034B | 100.00 | NHS Shropshire, Telford and Wrekin ICB - M2L0M |
| Rutland 004C | 100.00 | NHS Leicester, Leicestershire and Rutland ICB - 03W |
| Forest Heath 004B | 100.00 | NHS Suffolk and North East Essex ICB - 07K |
| East Riding of Yorkshire 031D | 100.00 | NHS Humber and North Yorkshire ICB - 02Y |
| Huntingdonshire 004E | 100.00 | NHS Cambridgeshire and Peterborough ICB - 06H |
| Herefordshire 019A | 99.99 | NHS Herefordshire and Worcestershire ICB - 18C |
| Rutland 001D | 99.99 | NHS Leicester, Leicestershire and Rutland ICB - 03W |
| Huntingdonshire 004A | 99.99 | NHS Cambridgeshire and Peterborough ICB - 06H |
| Cornwall 007E | 99.99 | NHS Cornwall and the Isles of Scilly ICB - 11N |
| Cornwall 026D | 99.99 | NHS Cornwall and the Isles of Scilly ICB - 11N |
| East Riding of Yorkshire 039C | 99.99 | NHS Humber and North Yorkshire ICB - 02Y |
| East Riding of Yorkshire 039B | 99.99 | NHS Humber and North Yorkshire ICB - 02Y |
| East Riding of Yorkshire 039D | 99.99 | NHS Humber and North Yorkshire ICB - 02Y |
| Breckland 011A | 99.99 | NHS Norfolk and Waveney ICB - 26A |
| South Somerset 007B | 99.98 | NHS Somerset ICB - 11X |
| Herefordshire 019C | 99.98 | NHS Herefordshire and Worcestershire ICB - 18C |
| Suffolk Coastal 002B | 99.98 | NHS Suffolk and North East Essex ICB - 06L |
| Forest Heath 008C | 99.98 | NHS Suffolk and North East Essex ICB - 07K |
| Cheshire East 032C | 99.98 | NHS Cheshire and Merseyside ICB - 27D |
| Cornwall 026A | 99.98 | NHS Cornwall and the Isles of Scilly ICB - 11N |
| Forest Heath 008B | 99.98 | NHS Suffolk and North East Essex ICB - 07K |
| Cornwall 009A | 99.98 | NHS Cornwall and the Isles of Scilly ICB - 11N |
| Forest Heath 004E | 99.97 | NHS Suffolk and North East Essex ICB - 07K |
| Carlisle 001B | 99.97 | NHS North East and North Cumbria ICB - 01H |
| South Somerset 004A | 99.97 | NHS Somerset ICB - 11X |

LSOA = Lower Super Output Area

## Table S13. CCGs percent difference in monthly prescription rates of oral anticoagulants medication between March 2020 – April 2022. Top 10 CCGs with the highest and lowest percentage difference.

| **Lowest percent difference** | |
| --- | --- |
| **CCG Name** | **Percent difference^1^** |
| NHS Bristol, North Somerset and South Gloucestershire CCG | -9.75% |
| NHS South East Staffordshire and Seisdon Peninsula CCG | -8.65% |
| NHS Herts Valleys CCG | -8.41% |
| NHS Morecambe Bay CCG | -7.04% |
| NHS South Sefton CCG | -6.68% |
| NHS East Riding of Yorkshire CCG | -6.51% |
| NHS East Staffordshire CCG | -6.39% |
| NHS Chorley and South Ribble CCG | -6.36% |
| NHS Heywood, Middleton and Rochdale CCG | -6.31% |
| NHS Nottingham and Nottinghamshire CCG | -6.2% |
|  |  |
| **Highest percent difference** | |
| **CCG Name** | **Percent difference^1^** |
| NHS Bradford District and Craven CCG | 5.46% |
| NHS North East Essex CCG | 1.88% |
| NHS Blackburn with Darwen CCG | 0.24% |
| NHS Calderdale CCG | 0.18% |
| NHS Kernow CCG | -0.23% |
| NHS Fylde and Wyre CCG | -0.3% |
| NHS Newcastle Gateshead CCG | -0.47% |
| NHS Somerset CCG | -0.7% |
| NHS Oldham CCG | -1% |
| NHS Cannock Chase CCG | -1.05% |

^1^ Monthly percentage difference of prescription rate was calculated using the formula: (observed rate – expected rate) / expected rate × 100%, which was then averaged over March 2020 – April 2022.

## Table S14. CCGs percent difference in monthly prescription rates of hypertension and heart failure medication between March 2020 – April 2022. Top 10 CCGs with the highest and lowest percentage difference .

| **Lowest percent difference** | |
| --- | --- |
| **CCG Name** | **Percent difference^1^** |
| NHS Herts Valleys CCG | -7.11% |
| NHS South East Staffordshire and Seisdon Peninsula CCG | -6.15% |
| NHS South Sefton CCG | -5.78% |
| NHS Basildon and Brentwood CCG | -5.53% |
| NHS Salford CCG | -5.28% |
| NHS Bristol, North Somerset and South Gloucestershire CCG | -5.06% |
| NHS North West London CCG | -4.07% |
| NHS North Tyneside CCG | -3.95% |
| NHS North Lincolnshire CCG | -3.93% |
| NHS Morecambe Bay CCG | -3.85% |
|  |  |
| **Highest percent difference** | |
| **CCG Name** | **Percent difference^1^** |
| NHS Castle Point and Rochford CCG | 4.05% |
| NHS Southport and Formby CCG | 3.79% |
| NHS West Leicestershire CCG | 1.97% |
| NHS Vale of York CCG | 1.94% |
| NHS Somerset CCG | 1.88% |
| NHS Calderdale CCG | 1.15% |
| NHS Bradford District and Craven CCG | 0.94% |
| NHS Bassetlaw CCG | 0.83% |
| NHS Bedfordshire, Luton and Milton Keynes CCG | 0.48% |
| NHS Newcastle Gateshead CCG | 0.2% |

^1^ Monthly percentage difference of prescription rate was calculated using the formula: (observed rate – expected rate) / expected rate × 100%, which was then averaged over March 2020 – April 2022.

# Figures

## Figure S1: Observed and predicted prevalence of hypertension pre and post COVID-19 in England.


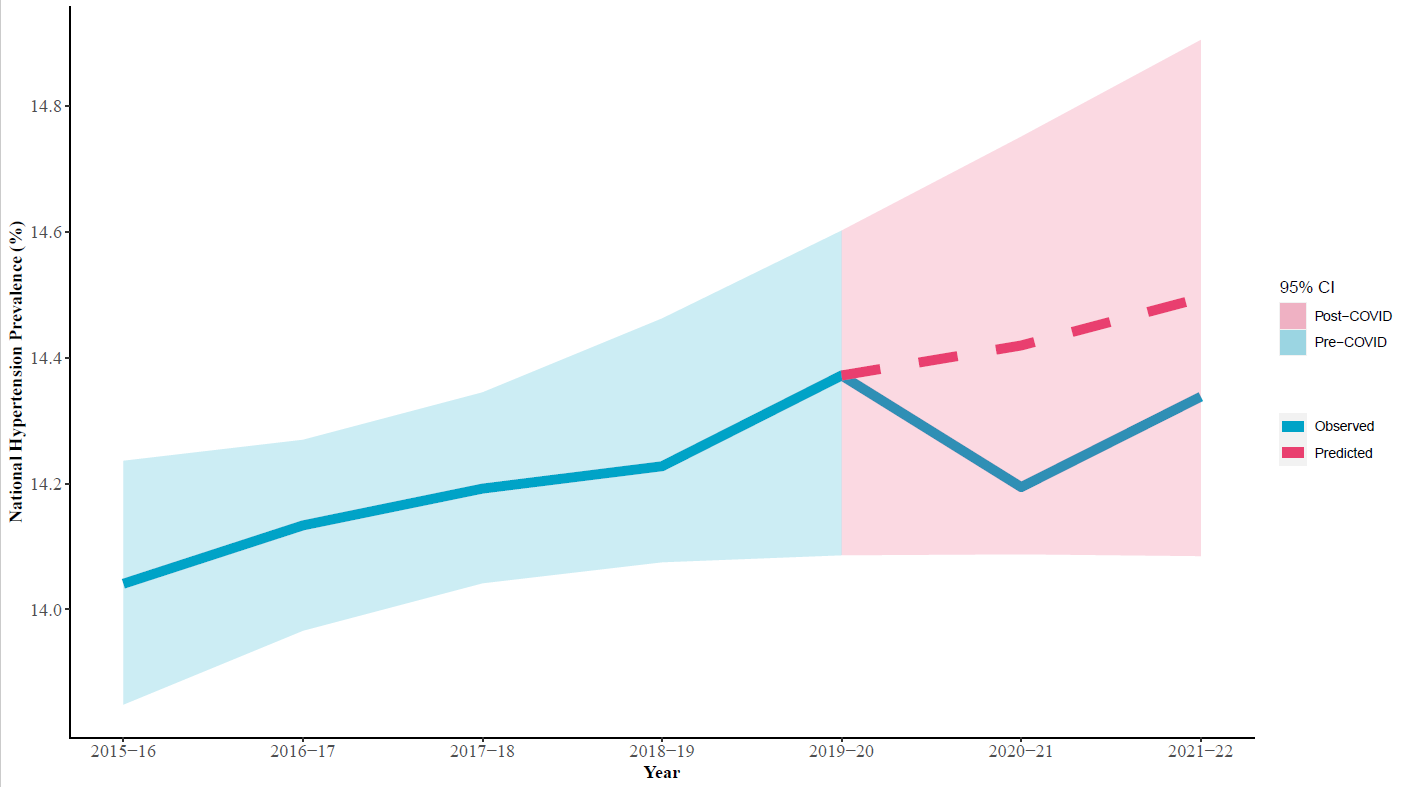


## Figure S2: Observed and predicted prevalence of atrial fibrillation pre and post COVID-19 in England.


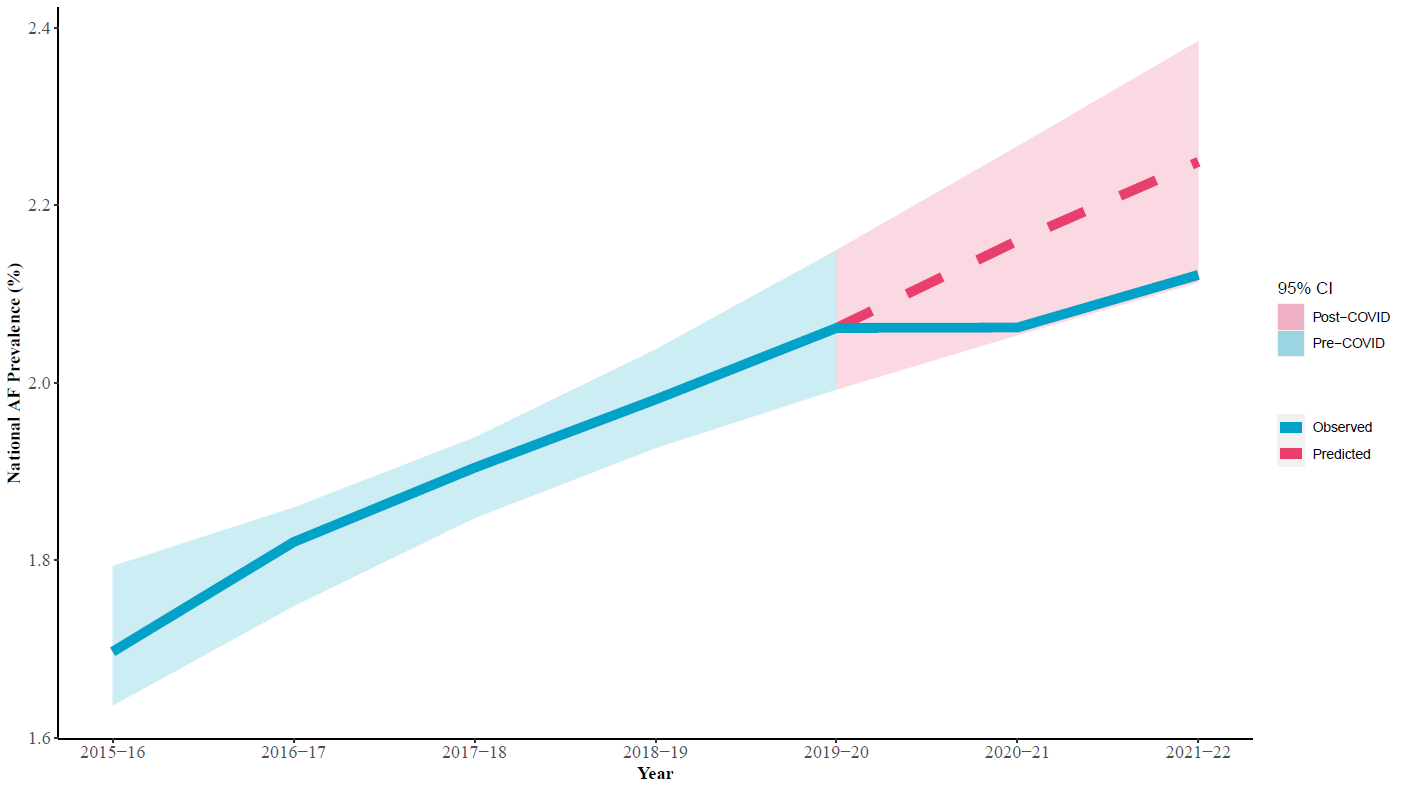


## Figure S3. Age standardised prevalence rates of A) Hypertension Figure S3. Missed diagnoses of atrial fibrillation between April 2020 and March 2021


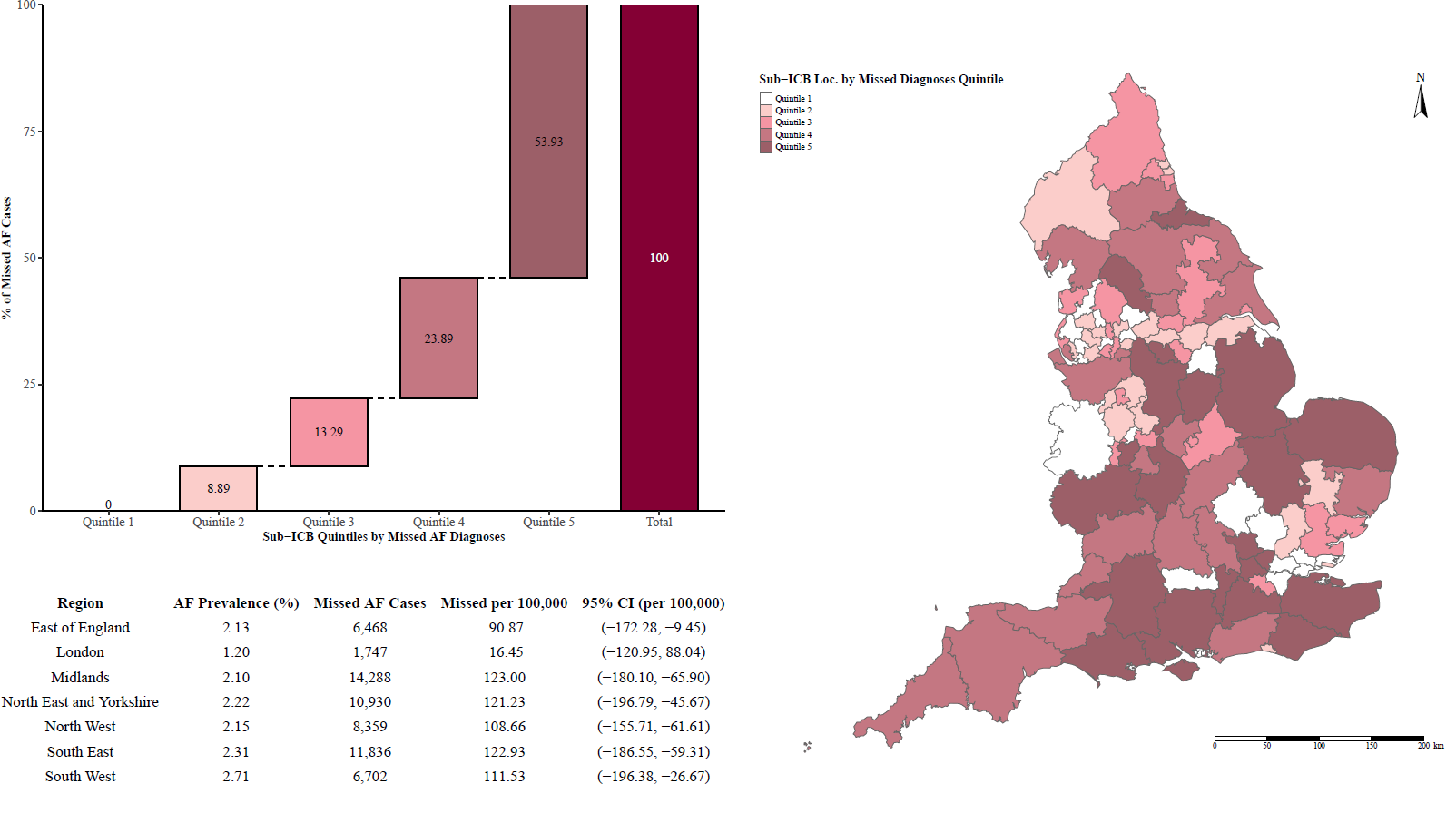


## Figure S4. Treatment achievement rates in 2019/20 and 2021/22 at sub-ICB level in England for A) Hypertension, B) individuals with a CHA2DS2-VASc score of 2 or more treated with anti-coagulation therapy and C) AF risk assessment

A.


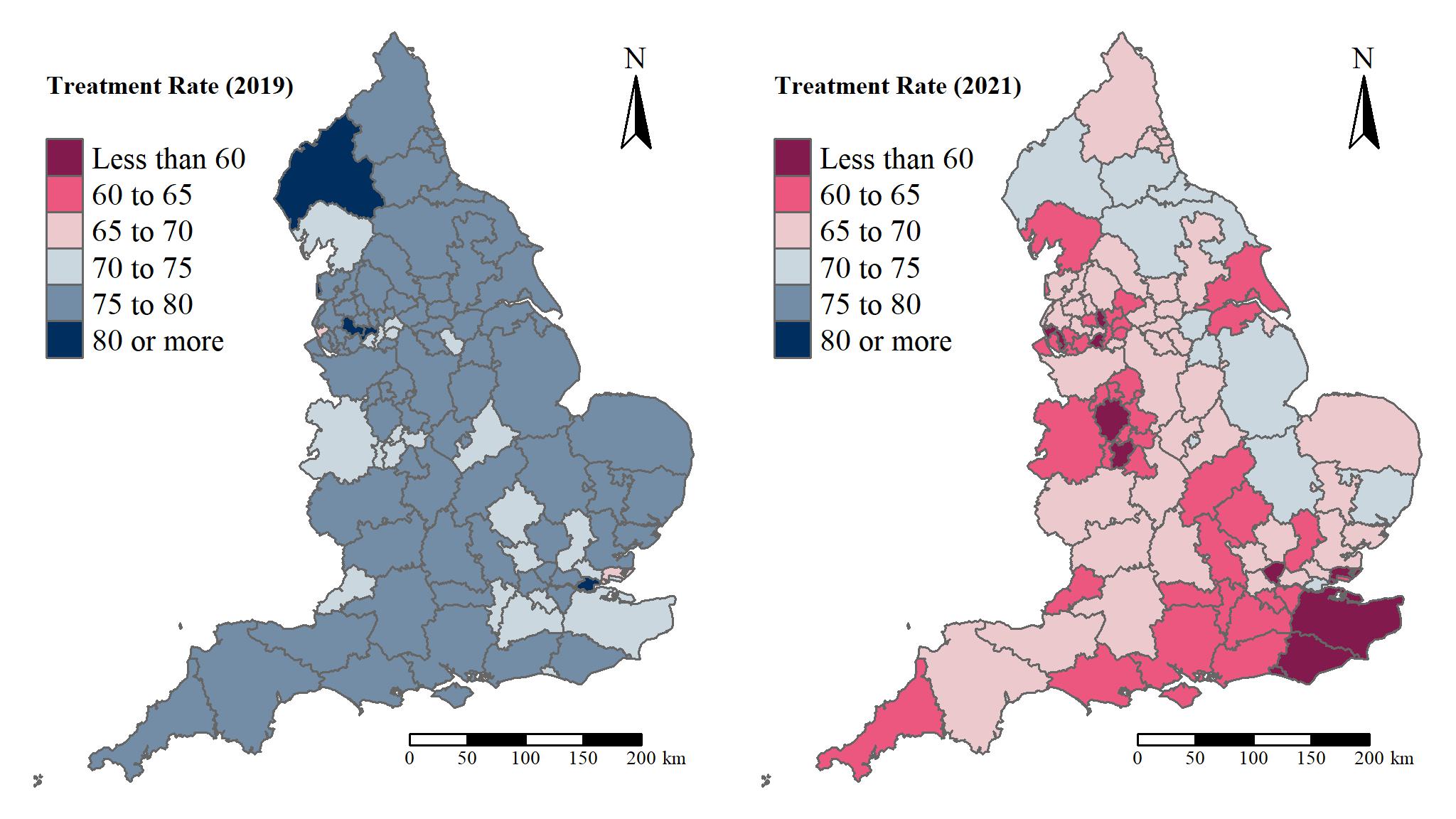


B.


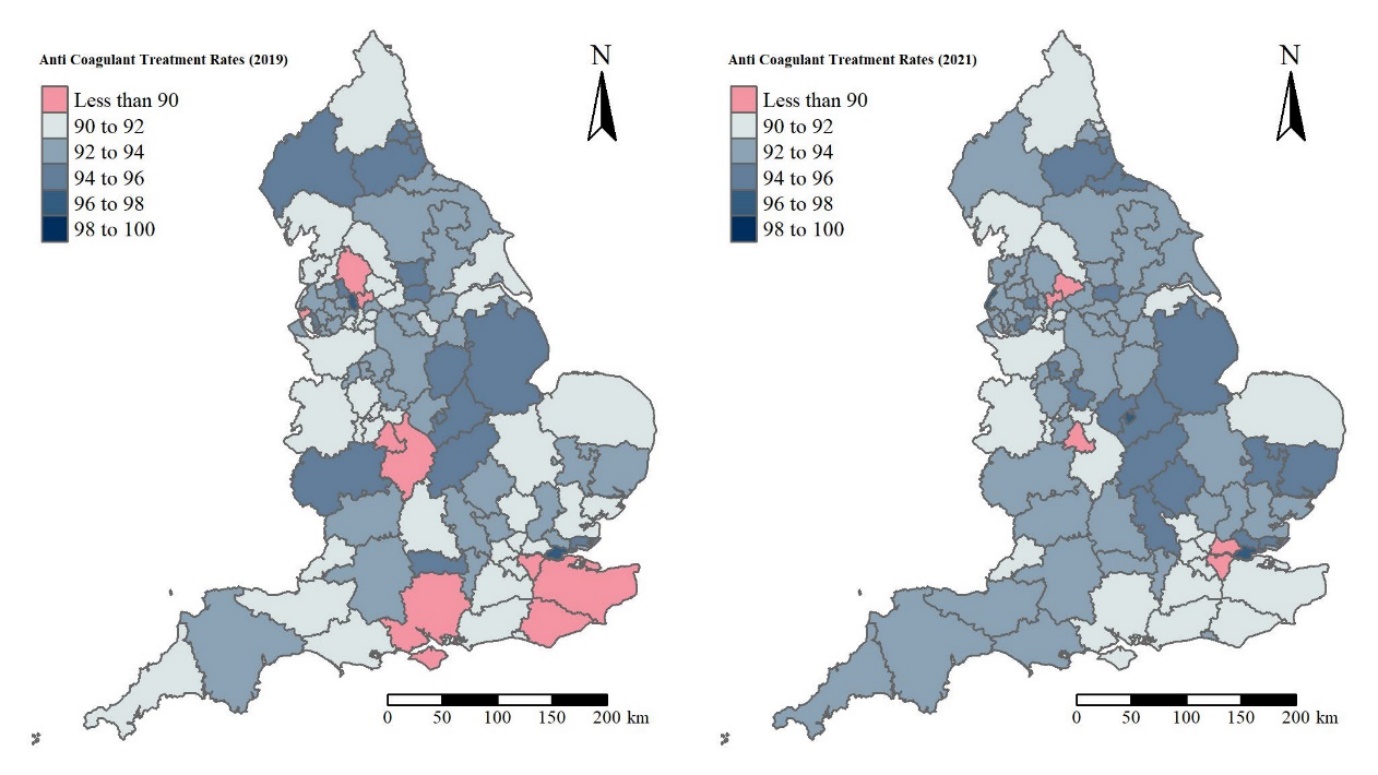


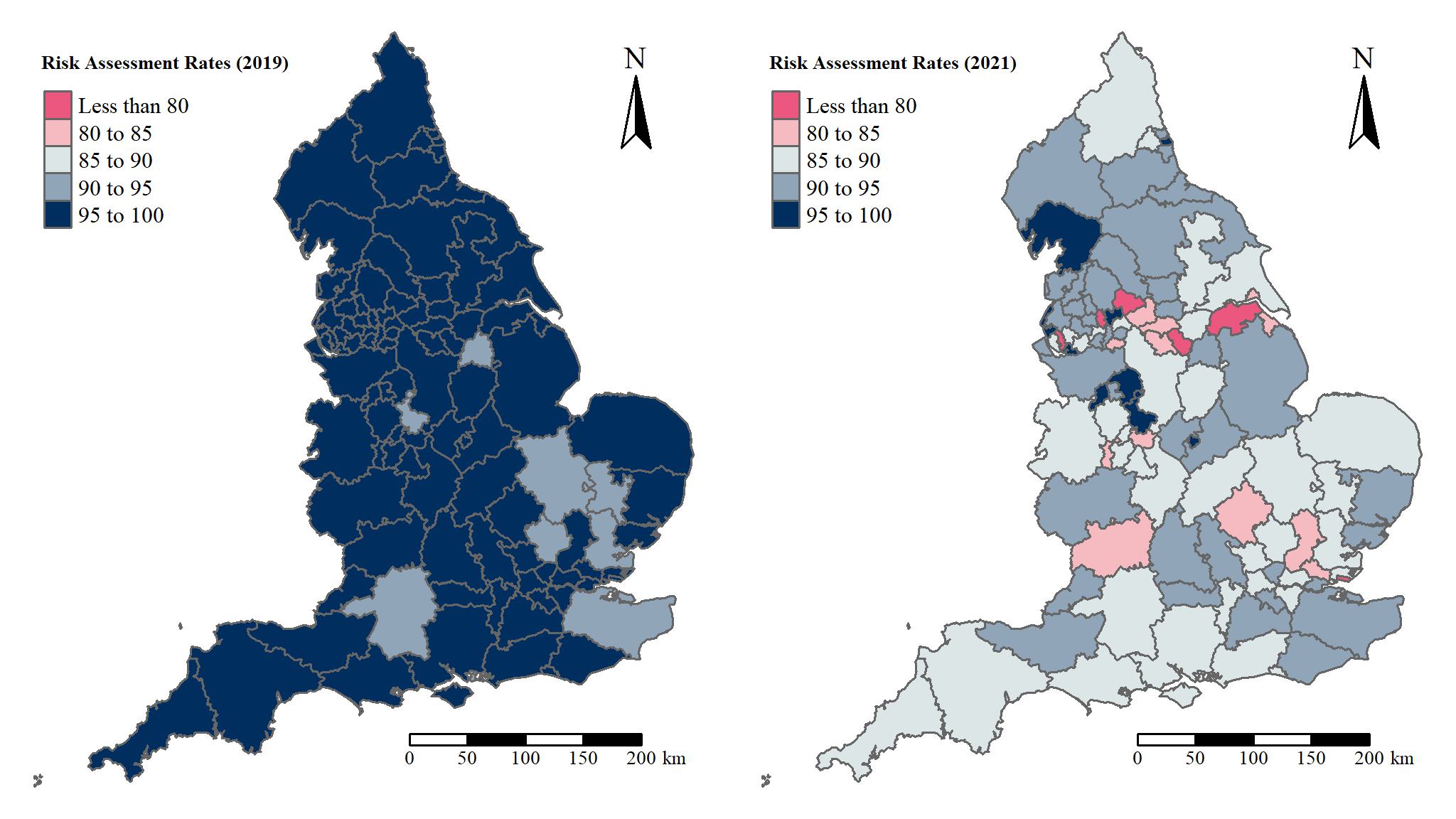


C.

## Figure S5. Prescription rate (items per 1,000 person days) for medications for A) Heart failure, B) Hypertension, C) both Hypertension and Heart Failure and D) Oral anticoagulants pre and post COVID-19.


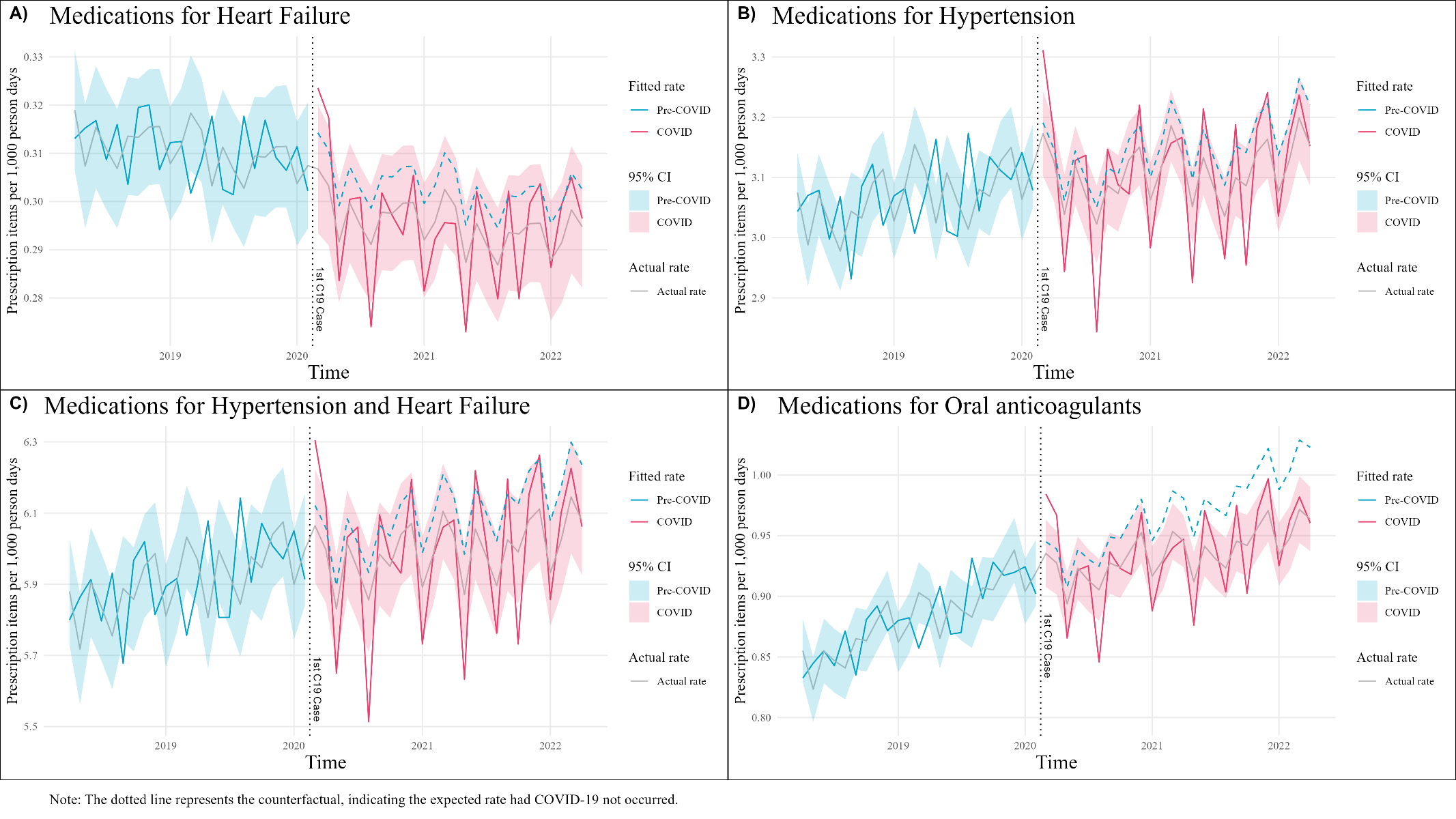


## Figure S6. Age standardised prevalence of Atrial fibrillation and hypertension by index of deprivation decile.


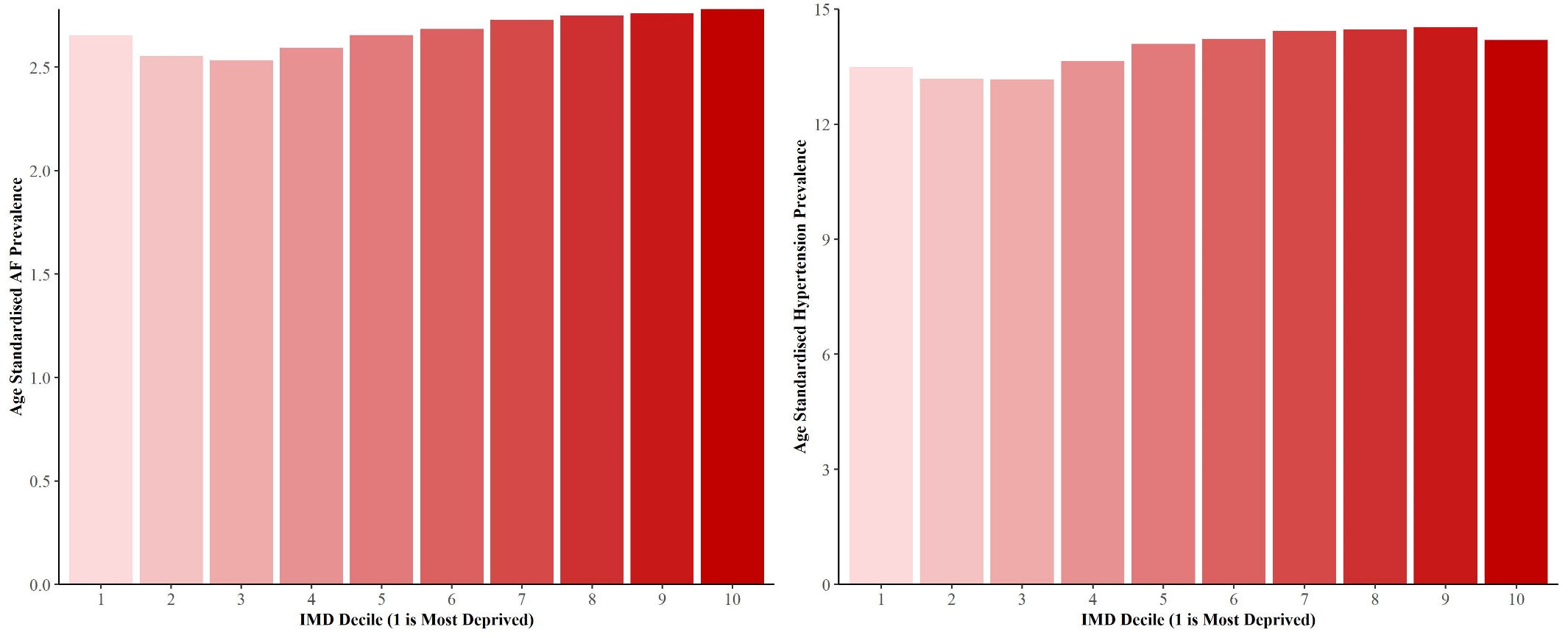


Note: Age standardised prevalence of hypertension in LSOAs in the most deprived decile was 13.55% compared to 14.16% in the least deprived decile (p<0.0001). Similar figures for AF were 2.65% and 2.78% respectively (p < 0.0001)

## Figure S7. Missed diagnoses of A) Hypertension and B) Atrial fibrillation by index of deprivation decile.


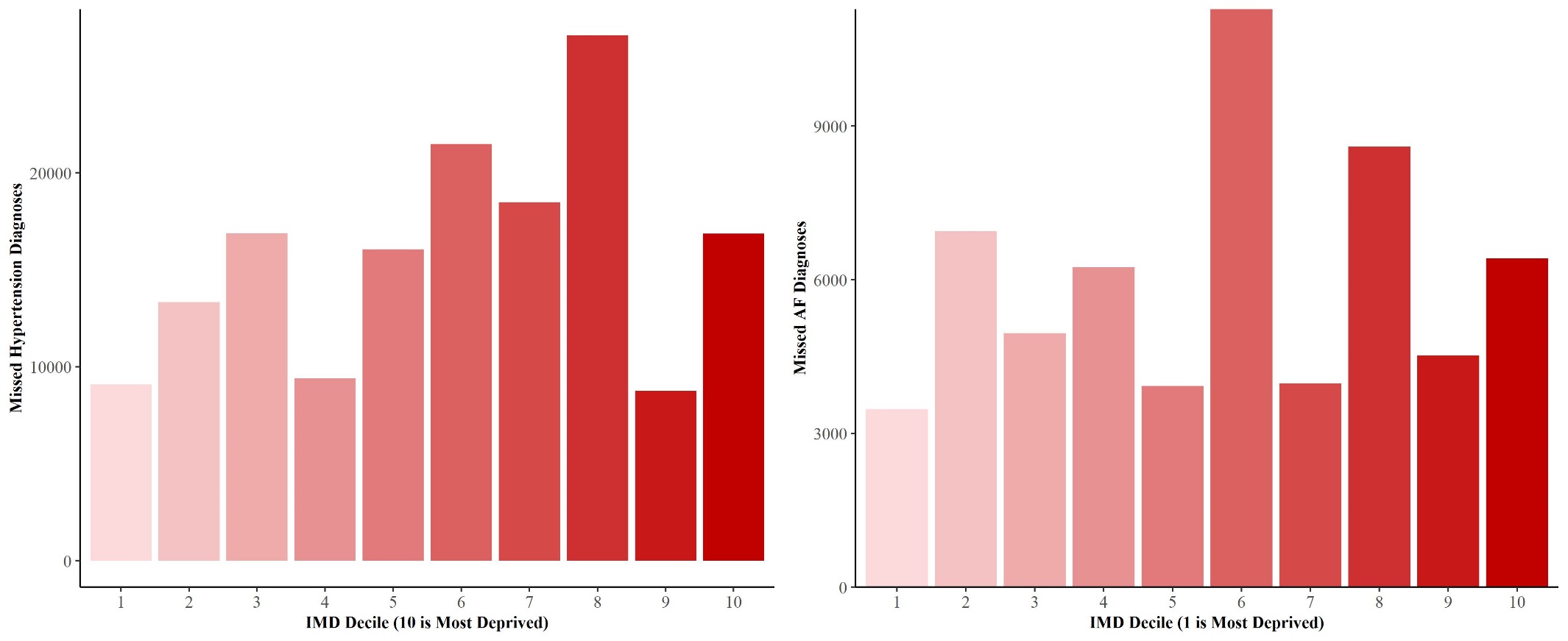


Note: No association between deprivation and missed diagnoses of hypertension and AF (p>0.05)

## Figure S7. Treatment achievement rates of A) Hypertension and B) Anti-coagulation and C)risk assessment by index of deprivation decile.


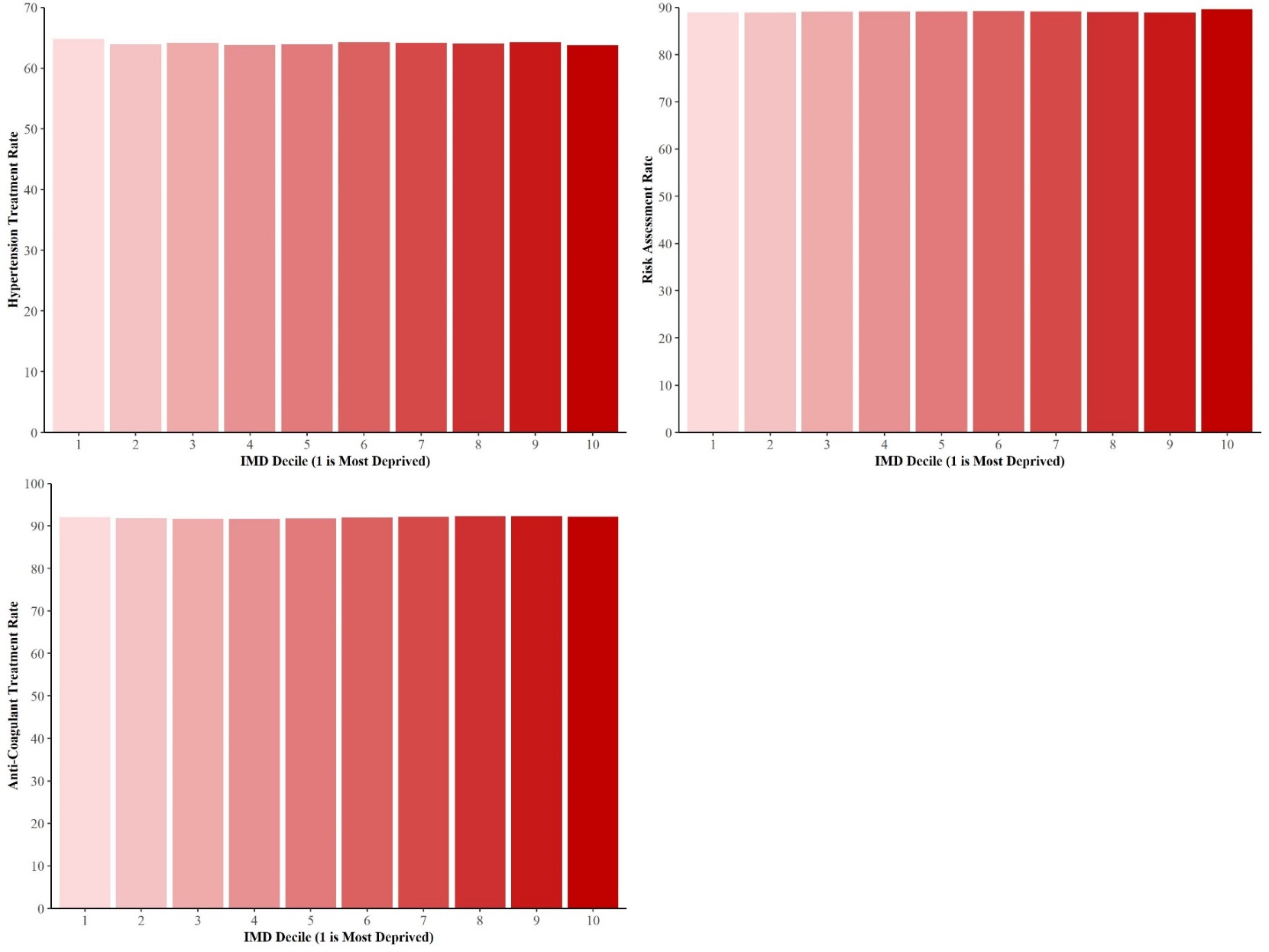


Note: The percentage difference between the most and least deprived areas although statistically significant for hypertension (<0.0015) and anti-coagulant treatment indicators (p<0.0001) was in absolute terms negligible
